# Systematically benchmarking peptide-MHC binding predictors: From synthetic to naturally processed epitopes

**DOI:** 10.1371/journal.pcbi.1006457

**Published:** 2018-11-08

**Authors:** Weilong Zhao, Xinwei Sher

**Affiliations:** Global Research IT, Merck & Co., Inc., Boston, MA, United States of America; La Jolla Institute for Allergy and Immunology, UNITED STATES

## Abstract

A number of machine learning-based predictors have been developed for identifying immunogenic T-cell epitopes based on major histocompatibility complex (MHC) class I and II binding affinities. Rationally selecting the most appropriate tool has been complicated by the evolving training data and machine learning methods. Despite the recent advances made in generating high-quality MHC-eluted, naturally processed ligandome, the reliability of new predictors on these epitopes has yet to be evaluated. This study reports the latest benchmarking on an extensive set of MHC-binding predictors by using newly available, untested data of both synthetic and naturally processed epitopes. 32 human leukocyte antigen (HLA) class I and 24 HLA class II alleles are included in the blind test set. Artificial neural network (ANN)-based approaches demonstrated better performance than regression-based machine learning and structural modeling. Among the 18 predictors benchmarked, ANN-based mhcflurry and nn_align perform the best for MHC class I 9-mer and class II 15-mer predictions, respectively, on binding/non-binding classification (Area Under Curves = 0.911). NetMHCpan4 also demonstrated comparable predictive power. Our customization of mhcflurry to a pan-HLA predictor has achieved similar accuracy to NetMHCpan. The overall accuracy of these methods are comparable between 9-mer and 10-mer testing data. However, the top methods deliver low correlations between the predicted versus the experimental affinities for strong MHC binders. When used on naturally processed MHC-ligands, tools that have been trained on elution data (NetMHCpan4 and MixMHCpred) shows better accuracy than pure binding affinity predictor. The variability of false prediction rate is considerable among HLA types and datasets. Finally, structure-based predictor of Rosetta FlexPepDock is less optimal compared to the machine learning approaches. With our benchmarking of MHC-binding and MHC-elution predictors using a comprehensive metrics, a unbiased view for establishing best practice of T-cell epitope predictions is presented, facilitating future development of methods in immunogenomics.

## Author summary

Computationally predicting antigen peptide sequences that elicit T-cell immune response has broad and significant impact on vaccine design. The most widely accepted approach is to rely on machine learning classifier, trained on large-scale major-histocompatibility complex (MHC)-binding peptide dataset. Because of the constant development of machine learning algorithms and expanding training data, providing comprehensive benchmarking of existing algorithms on blind testing dataset is important for recognizing the pros and cons of different algorithms and providing guidelines on specific applications. Here we present a study of such benchmarking by characterizing on a wide array of accuracy metrics, highlighting the best-in-class algorithms as well as their limitations. The rising concept that “naturally presented” antigen epitopes are more likely to generate effective T-cell immune response has led us to also consider the accuracy of these machine learning algorithms on predicting naturally presented peptides. We demonstrate that recent advance in incorporating high-quality naturally presented peptide data from mass spectrometry experiments has improved the accuracy. Our benchmarking of machine learning predictors for MHC-binding and MHC-naturally presented antigen peptides contributes to establishing best practice of computational T-cell epitope analysis, which also has implication in tumor neoantigen-based cancer vaccine discovery.

## Introduction

The increasing wealth of immunogenomic information generated by next-generation sequencing (NGS) technologies is boosting the application of cancer immunotherapy that takes full advantage of individual’s adaptive immunity by administrating personalized cancer vaccines.[1–3] An essential step in provoking adaptive immunity, delivered by the activated CD8+ or CD4+ T cells, is the recognition of T cell receptor (TCR) to T cell epitopes.[4] As sequence repertoire for potential TCR-recognizing epitopes, whole exome or transcriptome from pathogens or tumor cells can be analyzed by bioinformatics pipelines to identify vaccine candidates.[5,6] Among various processes related to antigen presentation, the binding of antigen peptides to MHC proteins is considered to be the major determinant. Therefore, computational predictors that identify MHC-binding peptides in a high-throughput fashion are critical.[7–9] In principle, these predictors utilize availability of the large-scale peptide-MHC binding affinity matrix from experimental measurements, to train machine learning (ML)-based classifiers to distinguish MHC-binders from non-binders.[10] While all serving the purpose of MHC-binding prediction in general, the increasing method variations among these tools, in combination with the emerging new types of experimental data, render it necessary to rationally select the best approach, especially for the potential applications in cancer vaccine design.

Immune Epitope Database (IEDB) hosts a series of ML-based tools, each trained on specific dataset of experimental peptide-MHC binding affinity matrix.[10] These different tools encompass two common approaches of ML (Table 1), namely, linear regression (LR) and artificial neural network (ANN). LR predicts peptide-MHC binding affinity by fitting the weight matrix that relates peptide sequence to end-point binding affinity value. Depending on the specific parameters used, such as whether regularization of weight matrix is included during training stage, tools utilizing LR can be further categorized into naïve position-specific scoring matrix (PSSM)[11] and stabilized matrix method (SMM)[12]. The inclusion of regularization terms, in general, helps to prevent overfitting of LR weight matrix on training set. For MHC class I epitope prediction, SMM is widely adapted, including smm[12], smmpmbec[13], and PickPocket[14]. For MHC class II, IEDB-hosted tools also contain those applying naïve PSSM, including comblib[15] and tepitope[16]. The applicability of LR approaches to predict MHC-binding relies largely on the assumption that the contribution of individual residues to the overall binding affinity is linear in nature. While it has been shown that certain types of amino acids are predominant at MHC-anchoring positions of peptide epitopes[17], the correlation between neighboring residues was also demonstrated to affect MHC-binding. Therefore, ANN presents as a better approach to capture the non-linear relationship between peptide sequence and MHC-binding affinity, compared to LR.[18,19] In ANN, the contribution of residue type of peptides to MHC-binding is simulated by one or more hidden layers.[20] These nodes essentially add extra features in addition to the input peptide sequences and are able to comprehend the contribution of intrapeptide residue-residue interactions to the binding affinity. IEDB tools utilizing ANN include ann (NetMHC3.4) and NetMHC4 for MHC class I prediction[18,21], and nn_align (NetMHCII2) for MHC class II prediction[7,22]. To overcome the low reliability resulted by a lack of sufficient training data for specific human leukocyte antigen (HLA) alleles that encode MHC proteins, ANN pan-allele tools have also been developed, such as NetMHCpan and NetMHCIIpan.[23] These approaches perform imputation to obtain MHC-binding affinity of untrained allele, on the basis of neighboring MHC bearing the highest sequence similarity. Pan-allele methods exhibited comparable classification accuracy between common HLA alleles and rare alleles that were not yet trained in allele-specific approaches.

**Table 1:** Accuracy metrics for all tested MHC-binding prediction tools.

	AUC (± err)	SRCC (± err)	VUS (± err)	SPE	R-squared
MHC Class I (9-mer)					
***smm***	0.856 ± 0.014	0.701 ± 0.020	0.498 ± 0.025	0.677	0.490
**smmpmbec**	0.857 ± 0.014	0.700 ± 0.020	0.503 ± 0.025	0.682	0.493
**ann(NetMHC3.4)**	0.860 ± 0.014	0.708 ± 0.019	0.537 ± 0.026	0.805	0.551
**NetMHC4**	0.881 ± 0.012	0.725 ± 0.017	0.545 ± 0.025	0.836	0.574
**PickPocket**	0.796 ± 0.016	0.580 ± 0.025	0.437 ± 0.022	0.332	0.339
**consensus**	0.860 ± 0.014	0.706 ± 0.019	0.521 ± 0.026	0.755	0.527
**NetMHCpan2.8**	0.846 ± 0.015	0.690 ± 0.020	0.519 ± 0.026	0.792	0.536
**NetMHCpan3**	0.880 ± 0.012	0.731 ± 0.016	0.545 ± 0.025	0.836	0.580
**NetMHCpan4**	0.872 ± 0.012	0.732 ± 0.016	0.536 ± 0.025	0.836	0.581
**NetMHCcons**	0.838 ± 0.018	0.675 ± 0.024	0.520 ± 0.029	0.810	0.530
**mhcflurry**	0.911 ± 0.010	0.761 ± 0.015	0.614 ± 0.026	0.813	0.641
**mhcflurry_pan**	0.873 ± 0.012	0.740 ± 0.016	0.549 ± 0.025	0.873	0.599
**MixMHCpred**	0.842 ± 0.021	Not calculated	0.399 ± 0.040	0.116	Not calculated
MHC Class II (15-mer)					
***NetMHCIIpan***	0.891 ± 0.005	0.784 ± 0.006	0.622 ± 0.011	0.326	0.579
**nn_align**	0.911 ± 0.004	0.838 ± 0.005	0.694 ± 0.011	0.577	0.671
**smm_align**	0.871 ± 0.006	0.746 ± 0.007	0.598 ± 0.011	0.256	0.537
**consensus**	0.851 ± 0.006	0.700 ± 0.008	0.556 ± 0.010	0.164	0.000
**comblib**	0.750 ± 0.010	0.499 ± 0.016	0.404 ± 0.015	0.158	0.102
**tepitope**	0.759 ± 0.010	0.522 ± 0.016	0.379 ± 0.011	0.237	0.186
**mhcflurry**	0.744 ± 0.007	0.495 ± 0.011	0.383 ± 0.010	0.060	0.238

Despite the cross-validation results reported previously for different approaches and tools, their prediction power is eventually determined by the performance on predicting “blind” dataset, that is, data that have never been exposed to the predictors. Benchmarking the trained predictors against blind peptide-MHC binding data can provide necessary metrics needed. While such effort has been attempted as an automated process on the IEDB server[19] (~ 7000 peptides across 42 HLA alleles with experimental IC50 available, since 2014), the evaluation metric reported only contains the ranking score of each tool, lacking other detailed metrics such as correlation of absolute binding affinities and accuracy of predicting strong binders, which are critical for precise epitope selection. Also, allele-specific accuracy is not reported. Furthermore, the benchmarking on emerging, high-quality mass spectrometry (MS) peptide is yet available. The same gaps also apply to the MHC class II benchmarking.[24] In this paper, we aim to deliver a systematic and quantitative benchmarking of a spectrum of MHC class I and II binding predictors, using blind binding affinity dataset collected from both IEDB consortium as well as independent studies.

With respect to the application of MHC-binding prediction in vaccine development, a significant gap of capacity is the lack of knowledge on the correlation between the MHC binding affinity and the immunogenicity of peptides.[25,26] While ML-based predictors are capable of selecting potent MHC-binders, the selected sequences can only be presented to TCR if they are truly generated by proteasome cleavage and transported to MHC within antigen-presenting cells (APCs).[27,28] The accuracy in predicting these two processes is limited by the volume of high-quality training set. For the same reason, accurately predicting immunogenic T cell epitopes by trained ML framework has also been a daunting task.[29] Previous attempts to filter epitope-based vaccine candidates by solely relying on MHC-binding prediction have discovered that a majority of predicted binders were non-immunogenic.[30,31] Therefore, evaluating the accuracy of different binding affinity predictors in identifying naturally processed T cell epitopes is of critical relevance to their applications in neoantigen and vaccine prescreening. Besides ML-based predictors, protein structural modeling taking advantage of high-resolution crystallographic data has emerged as an informative alternative, not only to predict peptide-MHC binding affinity, but also to guide understanding on the immunogenicity of peptide-MHC complex.[30,32] The development of structure-based prediction of peptide-MHC binding by peptide-protein docking algorithms has enabled enhanced sampling of peptide-protein binding landscape.[33,34] Structure-based predictions can potentially complement ML-based approaches by providing high-resolution peptide-MHC structure, which allows the further assessment on the TCR interaction and the immunogenicity of the predicted epitope. Hence, the structural modeling approach presents an opportunity for predicting the T cell epitope immunogenicity.

The aforementioned gaps in current knowledge formulate several key queries of this paper. We firstly introduced the test set and evaluate the prediction performance of MHC class I and II tools on the blind test set. The tools include published IEDB methods, MixMHCpred[17], and mhcflurry, as well as our development of pan-type I HLA epitope prediction version [20,35]. To provide a comprehensive understanding of prediction reliability, we performed evaluation metrics covering the prediction accuracy of binder classification and binding affinity ranking. We then benchmarked particular user cases, including understanding allele-specific performance and recapturing absolute binding affinity of strong MHC-binders. We also focused on evaluating the reliability of MHC-binding predictors to recover T cell-presenting epitopes using the dataset of naturally processed and eluted peptides. In addition, we demonstrated the performance of structure-based approach Rosetta FlexPepDock for peptide-MHC binding affinity prediction and explored its possible usage to improve T-cell epitope identification towards better identification power on T cell immunogenicity.

## Methods

### Datasets

#### Experimental binding affinities

A comprehensive dataset of MHC-binding peptidome was downloaded from IEDB database as of Oct. 2016.[36] The total number of entries is 513701. The most recent datasets used for training IEDB tools as well as mhcflurry was downloaded from IEDB.[37] For MHC class II, the “similarity reduced” set was selected. Several steps were taken to generate the test datasets for benchmarking prediction tools: Firstly, quantitative measurements were selected by choosing binding assay types that report K_D_, IC50, or EC50; secondly, the measurement values larger than 50000 nM were filtered out; thirdly, 9-mers for human HLA type I and 15-mers for human HLA type II alleles were chosen to be included in the test datasets; finally, the test sets were compared with training sets of MHC I and II prediction tools, and repetitive sequences in the test sets were removed to ensure that data in the test sets have not been exposed to prediction tools. 29 HLA type II alleles have not been included in the training of any prediction tools and therefore were discarded. In addition, two independent sets of binding affinity measurement for 9-mer peptides to HLA-B2705 and B3801alleles were also identified from literature and added to the MHC I cohort.[38,39] These steps resulted in two test datasets including 32 and 24 unique HLA type I and II alleles with 2827 and 15691 binding affinity values, respectively (Tables 2 and S1). MHC class I testing data are more heavily distributed in strong binder (IC50 < 50 nM) and non-binder (IC50 > 500 nM) regimes, while MHC class II are heavily distributed in non-binder (IC50 > 1000 nM) regime. These ratios are comparable to the existing training data. Compared to class I, more new data were generated for class II binding and non-binding peptides. 10-mer MHC-ligands from IEDB were preprocessed in the same fashion as the 9-mers, resulting in testing data comprised of 18 HLA type I alleles.

**Table 2:** Details of the blind test datasets included for current benchmarking.

Class	# of test data	# of HLA alleles included	% of strong binder (*IC50 < 50 nM*)	% of weak binder (*class I < 500 nM;* *class II < 1000 nM*)	% of non-binder (*class I > 500 nM;* *class II > 1000 nM*)	# of data trained by prediction tools*
MHC I (9-mer)	2827	32	38.8	11.6	49.6	43258
MHC I (10-mer)	747	18	50.9	11.2	37.9	22889
MHC II (15-mer)	15691	24	12.3	26.6	61.1	76763

*The number here accounts for the training datasets reported in Kim et al.(2014)[37] and Andreatta et al.(2011)[22], for MHC I and II respectively.

#### PDB structures of binding complexes

10 MHC I-peptide binding complexes were extracted from Protein Data Bank and were used as templates for the FlexPepDock modeling (S2 Table). The HLA-A and HLA-B alleles of MHC protein PDB entries have at least 50 unique peptide sequences and measurements in the test dataset to ensure a sufficient benchmarking between experimental and modeling results. For multiple PDB entries with the same allele, the model with highest resolution was used.

#### MHC Class I elution data

Three MS-derived elution datasets were introduced in the benchmarking:

- “Dana Farber”: The first elution dataset consisting of six HLA type I alleles was part of the test sets published in the *2nd Machine Learning Competition in Immunology 2012*, downloaded from Dana-Farber Repository.[40,41] This data include the elution property of each peptide as eluted (“+”) or negative (“-”) as verified by unpublished High-Performance Liquid Chromatography-MS experiments, and were widely employed in various previous efforts to predict naturally processed and MHC-bound peptides. For negative sequences, binding affinity assays have been performed to verify that they are MHC binders.
- “Abelin”: The second dataset originates from the MS-identified eluted peptidome on engineered mono-allelic HLA cell lines, published by Abelin et al.[42] For this dataset, we restricted benchmarking to top seven HLA types with the largest amount of associated peptides. For the “negative” class, 9-mer peptides were selected from the same proteomic database used by the authors for searching MS data. The selection was done in a similar fashion as Abelin et al., by randomly extracting 9-mer sequences and ensuring that no sequence can be aligned locally to the positive peptides. The number of negative peptides is at 1:1 ratio with the MS-confirmed positive ones for each HLA.
- “Sternberg”: The third dataset originates from MS-identified tumor antigen peptides in melanoma patients, published by Bassani-Sternberg et al.[43] The reported dataset has been pre-filtered with NetMHC4 to include only MHC class I binders and to select the strongest binding allele as target HLA type. In addition, we assigned the matching HLA types using the strongest binding affinity, predicted by NetMHC4 for each sample. We also restricted benchmarking to top seven HLA types with the largest amount of peptides associated. The “negative” class was generated using the same approach as in the previous Abelin dataset, with the exception that the positive to negative ratio was set to 1:50.

### MHC binding prediction tools

8 MHC Class I and 6 MHC Class II binding prediction methods hosted on IEDB Analysis Resource Server[10] were benchmarked (Table 3). Sequence submission was performed through RESTful API. mhcflurry[20], NetMHC4[18],NetMHCpan3[23], and MixMHCpred were locally installed on Linux server as stand-alone binary executables. NetMHCpan4 benchmarking was conducted on the website interface hosted by DTU Bioinformatics.

**Table 3:** Summary of ML-based prediction tools employed for current benchmarking.

MHC Class I binding predictor					
**Name**	**Method Principle**		**Details**		**Training Data Cutoff**
ann (NetMHC3.4)	ANN		2 to 10 hidden neurons; trained on 9-mer peptides		IEDB—2013
consensus	Combination		Value reported as the median of ann, smm, and PSSM		IEDB—2006
NetMHC4	ANN		5 hidden neurons; trained on all length peptides		IEDB—2014
NetMHCcons	Combination		Value reported as the best performer among NetMHC, NetMHCpan, and PickPocket		IEDB—2012
NetMHCpan2.8	ANN		Trained on 9-mer peptides; nearest neighbor searching for untrained allele		IEDB—2009
NetMHCpan3	ANN		56 or 66 hidden neurons; trained on all-mer length peptides		IEDB—2015
NetMHCpan4	ANN		Addition of MS-derived elution peptides to the training set and the prediction mode for elution probability score		IEDB—2017
PickPocket	LR		Alternative smm with binding specificity vectors of MHC pocket as additional features		IEDB—2009
smm	LR		SM with regularization term		IEDB—2005
smmpmbec	LR		smm + MHC binding pocket sequence		IEDB—2009
mhcflurry	ANN		32 or 64 hidden neurons; trained on 9-mer peptides		IEDB—2014
mhcflurry-pan	ANN		32 or 64 hidden neurons; trained on 43-mer peptides		IEDB—2014
MixMHCpred	Clustering + LR		Nearest neighbor clustering with distance calculated by PSSM		Collective HLA-peptidomics—2017
MHC Class II Binding Predictor					
Name		Method Principle		Details	Training Data Cutoff
nn_align (NetMHCII2)		ANN		2 to 60 hidden neurons; trained on 9-mer binding core with additional flanking region features	IEDB—2011
NetMHCIIpan		ANN		10 to 60 hidden neurons; trained on 9-mer binding core with additional flanking region features; nearest neighbor searching for untrained allele	IEDB—2014
consensus		Combination		Value reported as the median of nn-align, smm_align, and PSSM	IEDB -2010
smm_align		LR		SM with regularization term; trained on 9-mer binding core with additional flanking region features	IEDB—2007
comblib		LR		Naïve PSSM	IEDB—2008
tepitope		LR		Naïve PSSM with binding specificity of MHC pocket as additional features	IEDB—2001
mhcflurry		ANN		32 or 64 hidden neurons; trained on 15-mer all-length peptides	IEDB—2014

*PSSM (also know as Position-weighted Matrix): the binding specificity of each residue to a given MHC protein is represented by a score contributing independently to overall binding affinity. The derivation of position-specific score of individual amino acid is by regression method similarly applied in SM, but without the regularization term.

Based on the ML principle utilized, these tools can be divided into two general categories: LR-based binding score matrix and ANN approaches. PickPocket[14] is the only tool that is trained on features reflecting sequence space of MHC proteins rather than binding peptides. Pan-allele methods, including NetMHCpan2.8 and NetMHCpan3, utilize a nearest neighbor classification to assign untrained allele in quest to a trained allele based on similarity in binding pocket sequence. While other web-based prediction tools are not included in the current benchmarking, mainly due to lack of disclosed corresponding training datasets, their principles can be fitted into the aforementioned two types of ML approaches. Therefore, we are confident that the tools benchmarked in this study represent a comprehensive landscape of commonly applied MHC binding prediction algorithms.

### Details of mhcflurry_pan approach

**Fig 1:**
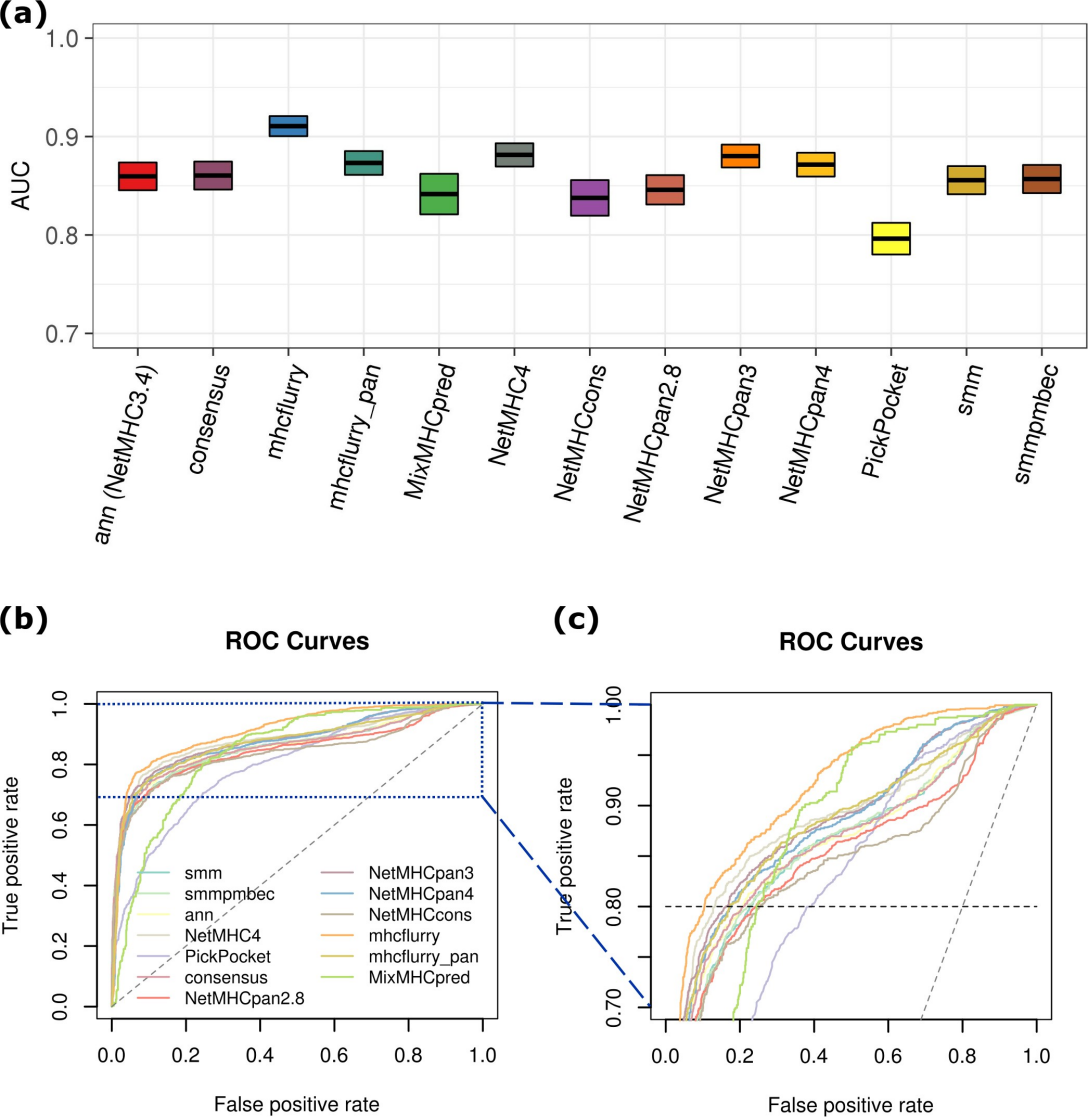
Binary classification (binder vs. non-binder) performance. (a) AUC of MHC-I binding epitope prediction tools. (b) ROC curves. IC50 = 500 nM was used as the cutoff for classifying experimentally measured epitopes. AUC was shown by box plot with upper and lower boundaries covering confidence level of 95%. (c) ROC curves enlarged for TPR between 0.7 and 1.0.

**Fig 2:**
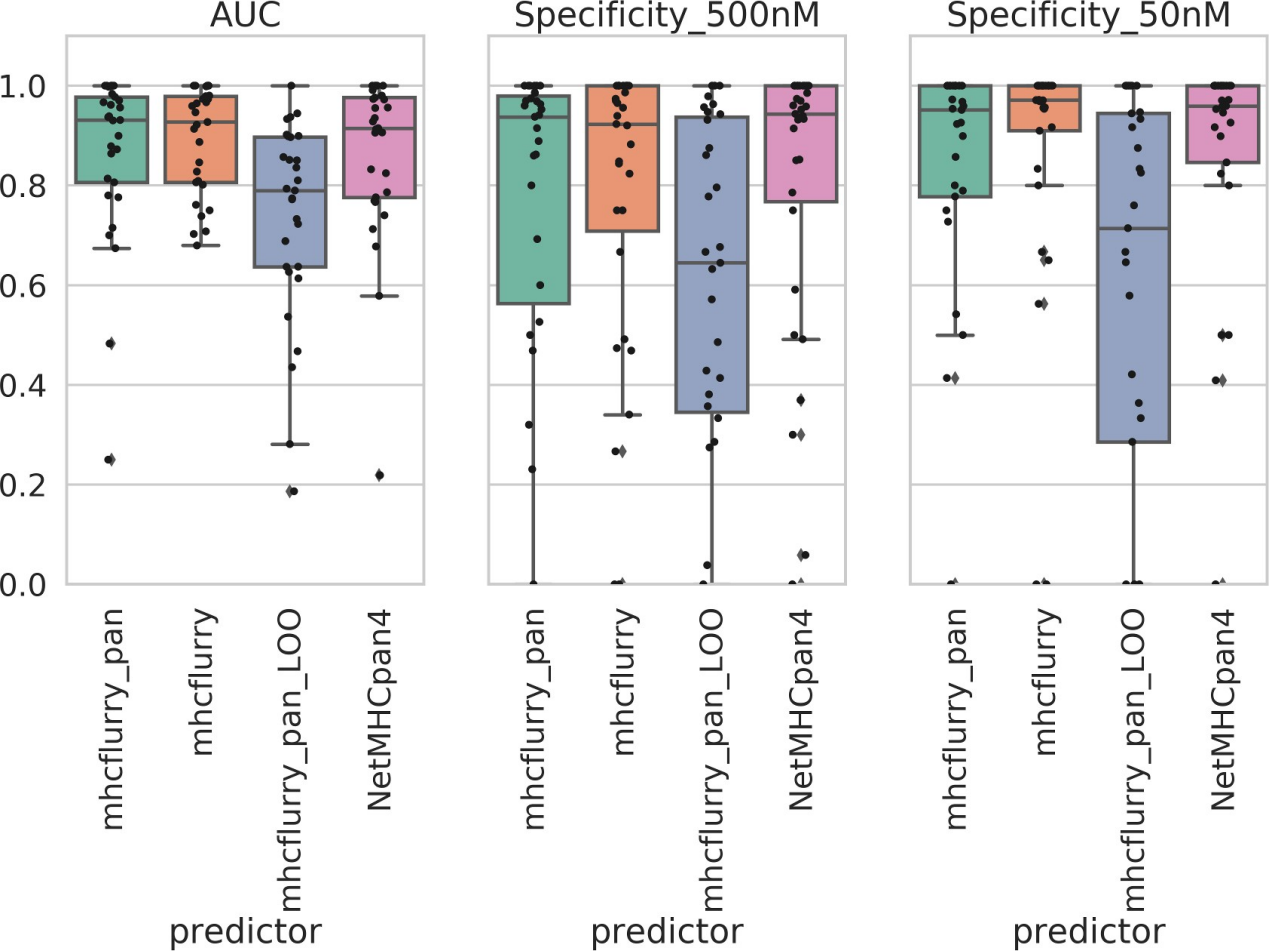
Evaluation of mhcflurry_pan predictor. Comparison of prediction power, indicated by (a) AUC, (b) specificity of binders, and (c) specificity of strong binders, of 9mer-based mhcflurry with 9mer-based NetMHCpan4, 43mer-based testing HLA included (mhcflurry_pan) and testing HLA leave-one-out (mhcflurry_pan_LOO) pan-predictor. Each point represents one HLA type.

Python package of mhcflurry has been pulled from the original code repository. The original version uses 9mer peptide as input feature with sparse matrix encoding of sequence. To incorporate the sequence of class I MHC, the input dimension has been extend to 43mer to create mhcflurry_pan (S6 Fig). The 34mer putative MHC binding pocket information was extracted from NetMHCpan3, which covers 3725 HLA types. Two branches of mhcflurry_pan were developed: 1) the modified ANN was trained on all available alleles of HLA binding data (mhcflurry_pan); and 2) the modified ANN was trained on all available alleles of HLA binding data, except leaving out the HLA type being tested (mhcflurry_pan_LOO). The accuracy of these approaches at both cross-allele and individual allele levels were benchmarked against mhcflurry (Figs 1 and 2). The final version of mhcflurry_pan is available at: https://github.com/juvejones/mhcflurry_pan.

### Rosetta FlexPepDock workflow

FlexPepDock protocol in Rosetta3.5[44,45] was implemented into the workflow of predicting binding of 9-mer peptides to MHC Class I proteins utilizing existing high-resolution crystal structure of peptide-MHC complexes. The protocol resembles the ones introduced previously with the addition of comparative modeling as the initial step for building bound peptide backbone based on template structure.[45,46] As a result, we were able to obtain a good conformational sampling of peptide with a low number of decoys (1000).[34] Starting from a peptide-MHC complex as the template, rotamer libraries of the peptide were firstly built, then incorporated for the following comparative modeling step. *Needle* package[47] was used to create the pairwise alignment profile between the target peptide sequence and the template required for comparative modeling. Through comparative modeling, backbone conformation of the target peptide was generated that resembles the template, providing a centroid-mode initial sampling of the peptide-MHC binding conformation.[46] The refinement step generated 1000 structures, which were optimized from the centroid-mode and provided high-resolution side chain packing structures within MHC pocket. Finally, clustering of the 1000 structures was performed based on 2.5 Å root-mean square distance (RMSD) cutoff. The lowest scoring model within the largest structural cluster was identified as the globally energy-minimized peptide-MHC binding complex. The reweighted scores, comprised of MHC protein energy, peptide energy, and peptide-MHC interfacial energy, were transformed into (0, 1) scale by establishing the highest and lowest scoring peptides in each allele as upper and lower boundaries, respectively. The resulted Rosetta Score for each peptide was aggregated and associated to the experimental binding affinity array. Visualization of the complex structures was made by VMD 1.9.3.[48]

### Evaluation metrics and statistical analysis

Receiver operating characteristic (ROC) curve and area under curve (AUC) were employed to benchmark the performance of binary classification between binders and non-binders, using a commonly applied cutoff of IC50 = 500 nM for MHC class I[49] and 1000 nM for class II[50]. Alternatively, we also evaluated HLA allele-specific IC50 cutoff based on established study of binding repertoire size, by using the specific IC50 value corresponding to identifying at least 75% of binder peptides of each HLA.[51] To further identify the performance of the prediction tools to distinguish strong binders from weak binders, volume under surface (VUS) was used to calculate the capacity to correctly classify a group of three peptides into strong binder, weak binder, and non-binder types on the basis of IC50 cutoffs of 50 and 500 nM:

$$VUS=\iint \{\mathrm{Pr}\left\{t_{-}\leq T\leq t_{+}\right\}\}d_{x}d_{y}$$

$$x=Pr\{T\leq t_{-}\}=F_{-}(t_{-}),\;y=Pr\{T>t_{+}\}=G_{+}(t_{+})$$

$$D_{00}=\{0\leq x\leq 1,\;0\leq y\leq G_{+}(F_{-}^{-1}(x))\}$$

where *t*_−_ and *t*_+_ indicate lower and upper cutoffs respectively, *Pr* indicates corresponding distribution probability, and D_00_ indicates integral space The calculation of VUS also introduces the specificity (SPE) measure of correctly assigning a peptide to strong binder.

Spearman’s ranking correlation coefficient (SRCC) between predicted and measured binding affinities was calculated to evaluate the reliability of binding predictions to correctly rank out stronger MHC-binding peptides. R-squared values were generated by linear regression of predicted IC50 to experimental IC50. Error estimation was performed based on 95% confidence interval. All analysis and corresponding data visualizations were implemented using R scripts. In particular, package ROCR[52] was used for ROC and AUC analysis and DiagTest3Grp[53] was used for VUS analysis. Note that MixMHCpred does not directly output binding affinity, so the ‘Max_score’ was used as the predicted affinity strength for calculating accuracy and correlations.

## Results

### MHC class I binding prediction tools

The half maximal inhibitory concentration (IC50) characterizes the effectiveness of a peptide in substituting a high affinity molecule for binding to MHC and represents the binding affinity of that peptide. The threshold of IC50 = 500 nM or 50 nM selects peptide binders or strong binders, respectively, to MHC and can be used to identify T cell epitopes. The accuracy of the predictors can thus be judged by the correctness of classifying peptides into binders and non-binders based on experimental results. For MHC I-peptide binding prediction, mhcflurry exhibits the best binary classification performance with AUC = 0.911 ± 0.010. ANN-based approaches with the most recent versions (mhcflurry, NetMHC4, NetMHCpan4, and NetMHCpan3) on average perform better than LR-based ones, including PickPocket, smm, and smmpmbec (Fig 1). Compared with LR, the ability of ANN to adapt weights of the hidden layer to capture the complex interactions between MHC-binding residues has been suggested previously.[43] In addition, ANN generally performs better on the task of regularization, leading to less overfitting on the training set. When comparing different versions of the same tool, newer versions (NetMHC4 and NetMHCpan3/4) generally outperform older ones (NetMHC3.4 and NetMHCpan2.8), with the exception of NetMHCpan4 versus NetMHCpan3. The improvement is likely a result of updated training set. On the other hand, since NetMHCpan4 was developed with the specific aim of improving the prediction of MS-derived, MHC-eluted peptides, the lack of better accuracy compared to NetMHCpan3 on binding affinity dataset is not surprising.

Note that AUC is independent of the cutoff chosen for binder versus non-binder classes, therefore providing the overall robustness with respect to accurately selecting MHC-binders from the peptide pool generated by all tumor somatic mutations. ROC curve also contains information about false-alarm rate in the positive class space defined by a given cutoff, which is the ratio of false positive rate (FPr) to true positive rate (TPr). For the high ANN performers such as mhcflurry and NetMHC4, high TPr (> 80%) can be achieved with relatively low FPr (~ 10%, Fig 1C). MixMHCpred demonstrates interesting behavior. While the overall AUC (0.842 ± 0.020) and the FPr at TPr = 80% (~ 22%) do not rank high among the methods, it attains second lowest FPr at TPr = 90%. As an important criterion for selecting reliable MHC-binding prediction, low FPr directly contributes to downsized and efficient experimental validation cycle. In this regard, mhcflurry represents a preferable tool as it achieves lowest FPr at either TPr = 80% or 90% (Fig 1B and 1C, orange), and MixMHCpred is also viable at TPr = 90% (Fig 1C, lime).

The preferable performance of mhcflurry encourages us to develop its pan-HLA version, mhcflurry_pan, on the basis of its open source ANN code (see Methods for details). Extended by the dimension of input sequence feature to 43mer, our version of mhcflurry_pan has achieved comparable predictive power of MHC-I epitopes in comparison with NetMHCpan4, with AUC = 0.873 ± 0.012 (Fig 1), with median AUC across alleles = 0.931 (Fig 2). Specificities of predicting both binder (< 500 nM) and strong binder (< 50 nM) are in the same statistical range with the original HLA-specific mhcflurry. We also tested the performance of mhcflurry_pan when trained by leave-one-out (mhcflurry_pan_LOO) approach, in which the corresponding binding data of HLA type in-test were not included. The accuracy of mhcflurry_pan_LOO maintained for a certain subset of alleles but lowered predictive power for others, with median AUC = 0.790. We note that this precision reaches similar level compared to the most recent version of NetMHCpan4.[54] Overall, mhcflurry_pan has achieved sound accuracy to facilitate the prediction of epitopes on untrained class I HLA types, which is critical for the study of tumor immunogenicity and design of vaccine epitopes across broad cancer patient populations.

**Fig 3:**
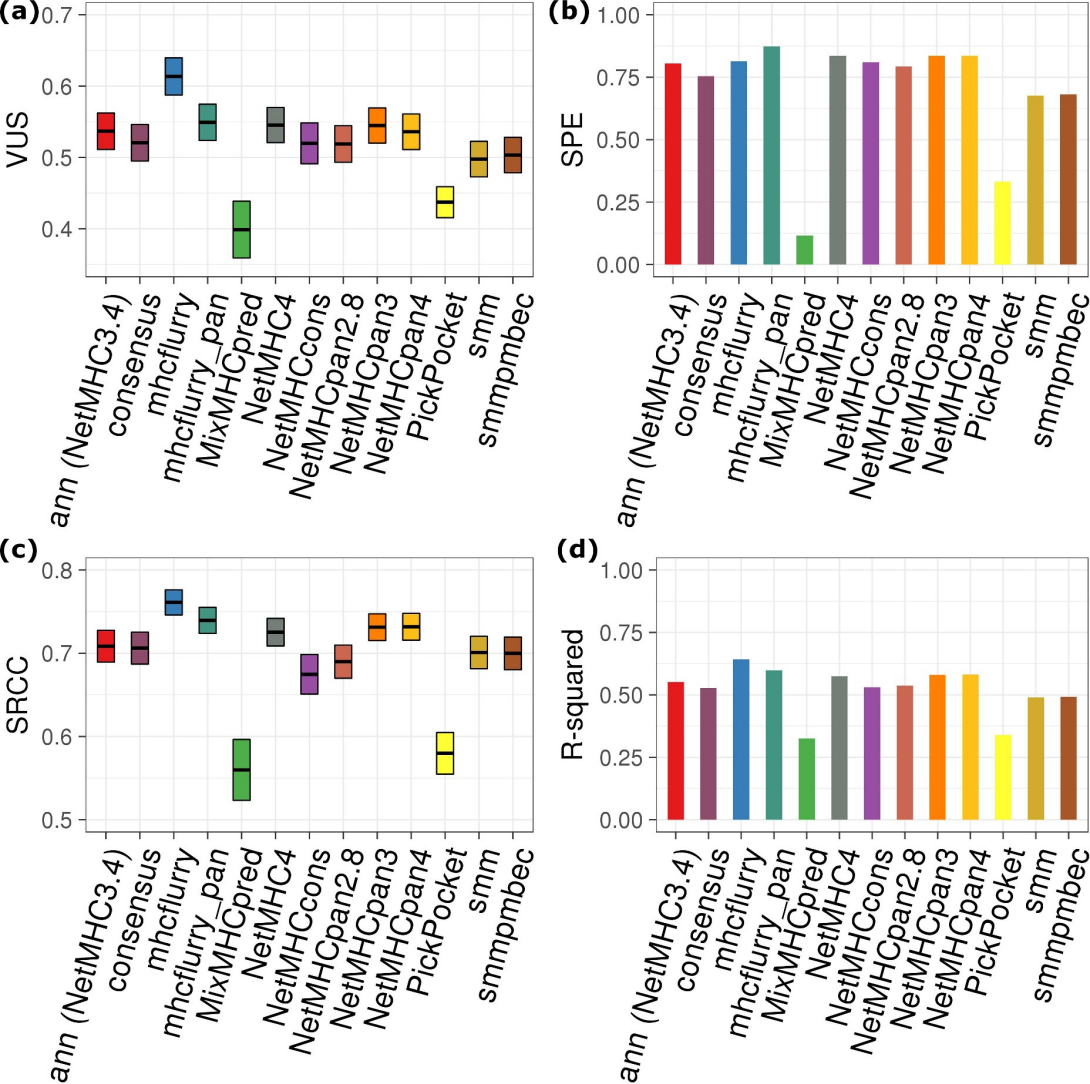
**Multiclass classification performance of MHC class I binding epitope prediction tools**: (a) VUS; (b) SPE; (c) SRCC; (d): R-squared of linear regression. IC50 thresholds of 50 nM and 500 nM were used to classifying experimental measurements between strong binder, weak binder, and non-binder. The box plots of VUS and SRCC show values covering 95% confidence level. Note that IC50 is not calculated in MixMHCpred.

In addition to conventionally used AUC criteria for differentiating MHC binder from non-binder, we introduce VUS (Volume Under Surface) as a measure for an additional classification of strong MHC binders that have IC50 < 50 nM (see Methods for details). This cutoff has been used empirically to further filter out peptides that may detach from MHC under instable thermal conditions. Fig 3 shows that mhcflurry still outperforms others with respect to three-class classification, followed by mhcflurry_pan and NetMHC4/NetMHCpan3. The ability of these tools to identify strong binders is also illustrated by specificity for predicting peptides with < 50 nM affinity (SPE, Fig 3B). For this benchmarking, mhcflurry_pan has the highest value 87.3% TPr on identifying peptides that have binding affinity < 50 nM. NetMHC4, NetMHCpan3, and NetMHCpan4 all have the second highest value (0.836).

For predicting the affinity ranking of peptides to MHC, we calculated SRCC and R-squared for the value pairs of experimental-predicted (Fig 3C and 3D). SRCC was employed commonly in previous studies to measure the correlation of affinity ranking between predictions and experimental values, proven valuable in selecting vaccine candidate epitopes. In addition to SRCC, we also calculated R-squared here to benchmark how close predicted affinities match experimental data. Both data indicate mhcflurry delivers the best correlation between predicted and experimentally measured binding affinities. A SRCC value of 0.761 ± 0.015 for mhcflurry demonstrates that mhcflurry is reliable with respect to ranking strong binders above weak binders when applied to epitope identification. On the other hand, the linear correlation between the predicted and measured absolute binding affinities, as indicated by R-squared, may not be satisfying. Even the best-in-class mhcflurry (R-squared = 0.641) can predict experimentally IC50 correctly only to a certain extent. Since the threshold for designating strong MHC binder peptides are only arbitrarily chosen at either 50 or 500 nM, the deficiency of these ML-based predictions tools at providing correct absolute binding affinity is likely to impact the identification of MHC-binding epitopes. A further confounding factor is that these threshold values were shown to be only applicable to certain alleles, while alleles with varied population frequency exhibiting different affinity ranges of binding repertoire.[51] While our data show that allele-specific cutoff generally does not alter the prediction accuracy in terms of AUC (S2 Fig), this still impacts the way to interpret true or false prediction rate at specific cutoff. The correct prediction of absolute peptide-MHC binding affinity is equally, if not more, significant compared with the binary classification task.

For MixMHCpred, the prediction score does not directly correspond to binding affinity due to the different type of HLA-ligandome training data. Therefore, the comparison with other predictors on SRCC and R-squared is not straightforward. In addition, the cut-point between ‘strong’ and ‘weak’ HLA-binders is not well-defined for MixMHCpred training data, leading to declined VUS and SPE (Table 1). Nonetheless MixMHCpred appears to be a valid candidate for binary classification of MHC-ligand.

Figs 1 and 3 demonstrate that mhcflurry is a superior choice for predicting 9-mer MHC I-binding epitopes, which is also in accordance with recent automated benchmarking results hosted on IEDB server.[19] We note that one advantage of mhcflurry for end-users is the Python API that enables tunable ANN training parameters, such as number of hidden neurons and dropout probability. In particular, setting dropout probability allows user-defined ANN to prevent overfitting on the training set. In this benchmarking, a relatively larger number of hidden neurons (64) and dropout rate of 0.1 were assigned to capture effectively the non-linear weight matrix relating 9-mer peptide sequence to binding affinity at a low degree of overfitting.

### MHC class I predictions of different peptide length

**Fig 4:**
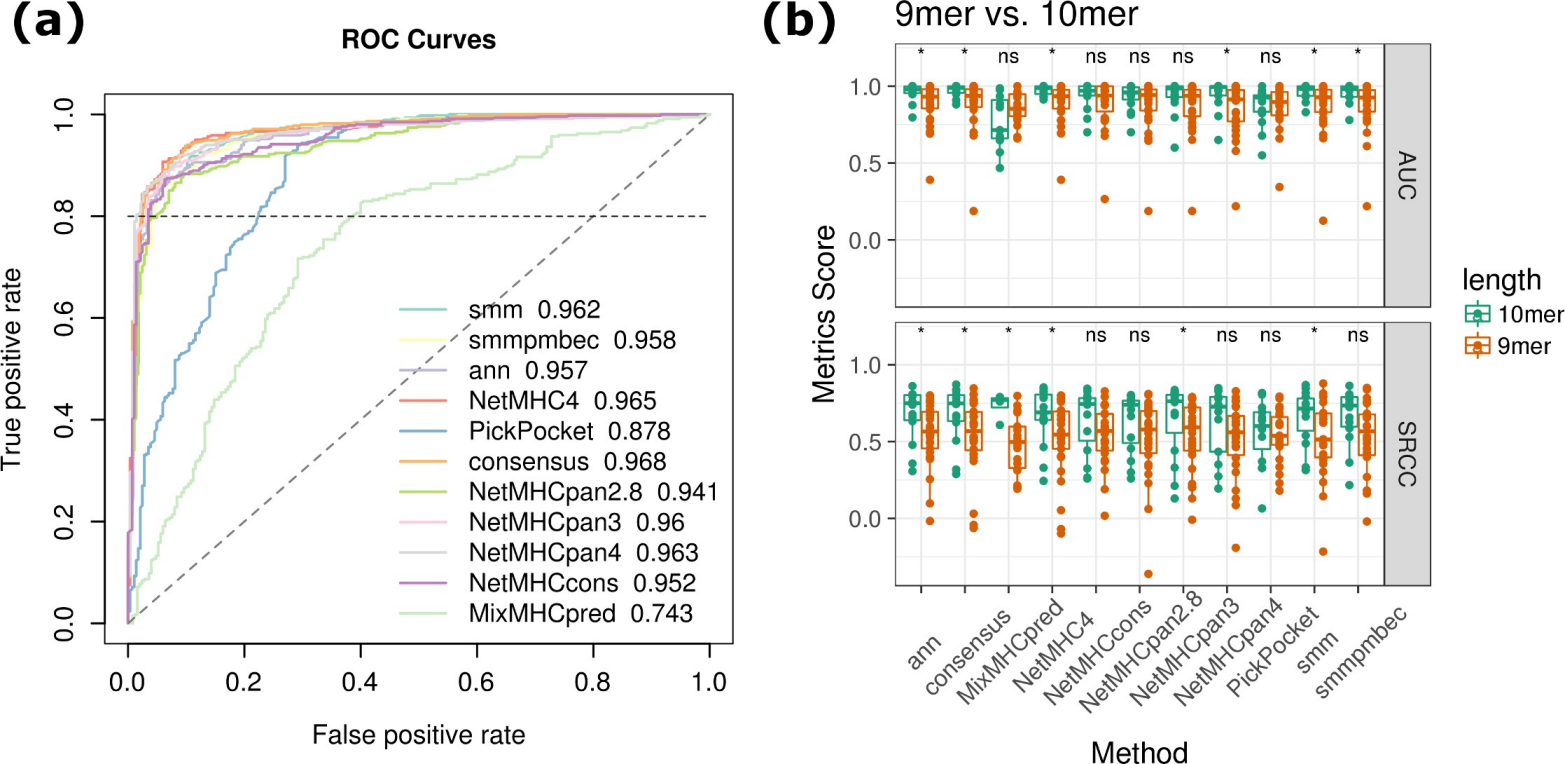
Comparison of prediction accuracy between 9-mer and 10-mer testing data. (a) ROC curves of 10-mer predictions with AUC value shown after each method. (b) Boxplots of AUC and SRCC calculated for 9-mer and 10-mer predictions, with each point representing a type I HLA allele. Significant levels were obtained by Wilcoxon test (*: p < 0.05; ns: p > 0.05).

While most MHC class I binding peptides are 9-mers, the length of MHC class I ligands may vary (3.4% 8-mer, 44.4% 9-mer, 29.9% 10-mer and 28.7% longer in IEDB database). Therefore, we also conducted analysis to compare the accuracy of class I MHC-ligand predictors between 9-mer and 10-mer testing data. All methods benchmarked for 9-mer dataset were considered expect mhcflurry and mhcflurry_pan. The highest AUC was obtained on consensus (0.968) and the second highest one was obtained on NetMHC4 (0.965). Overall the classification accuracy is encouraging as reflected by the ROC curves (Fig 4A). Among the 11 methods, six even demonstrated statistically significant higher accuracy for 10-mers than corresponding testing data for 9-mers (Fig 4B). Similar observation can be made on SRCC result as well. Taken AUC and SRCC together into consideration, NetMHC3.4, NetMHC4, smm, and consensus methods demonstrated consistently more reliable prediction for the HLA alleles tested.

### MHC class II binding prediction tools

**Fig 5:**
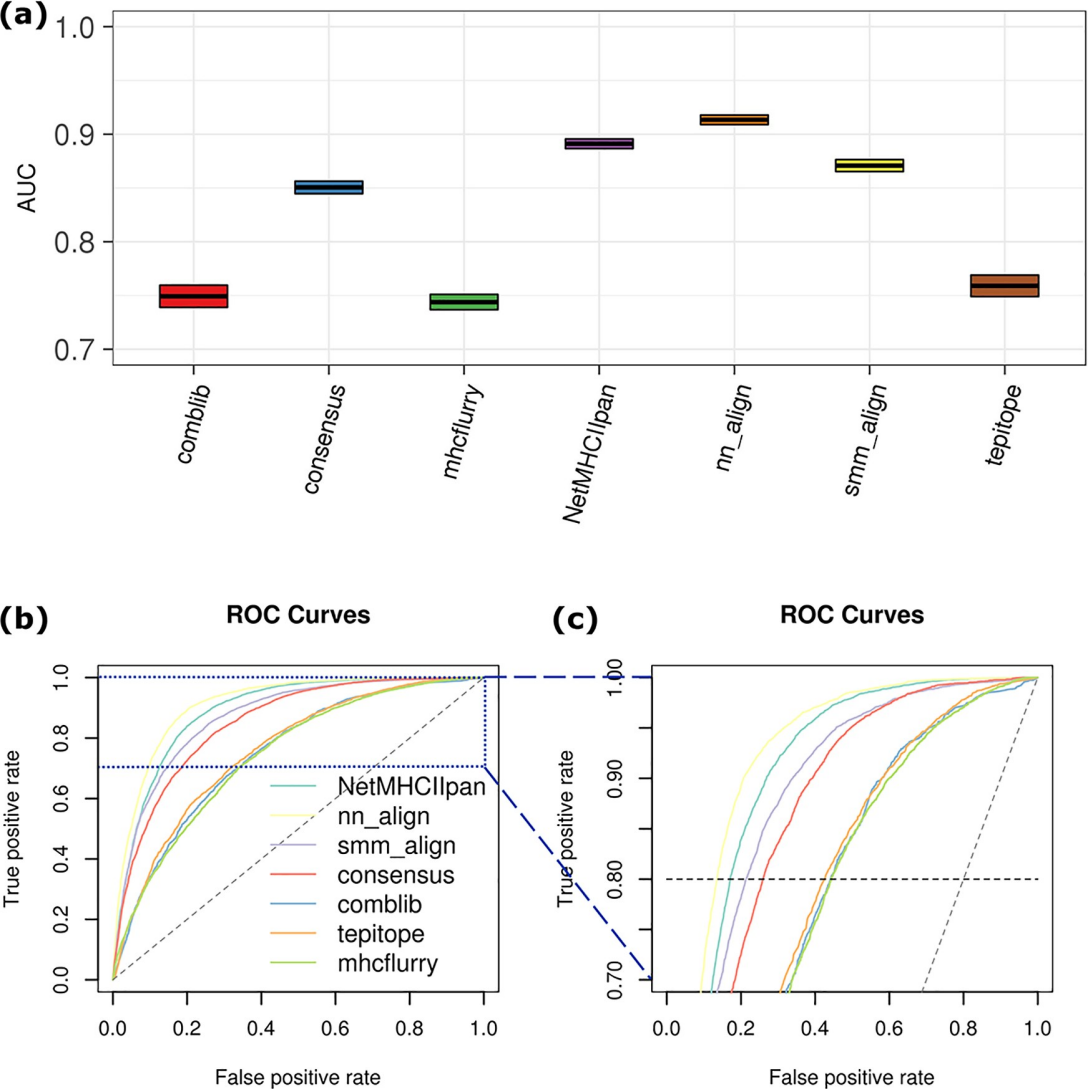
Binary classification performance of MHC-II binding epitope prediction tools. (a) AUC. (b) ROC curves. IC50 = 1000 nM was used as the cutoff for classifying experimentally measured epitopes. AUC was shown by box plot with upper and lower boundaries covering confidence level of 95%. (c) ROC curves enlarged for TPR between 0.7 and 1.0.

**Fig 6:**
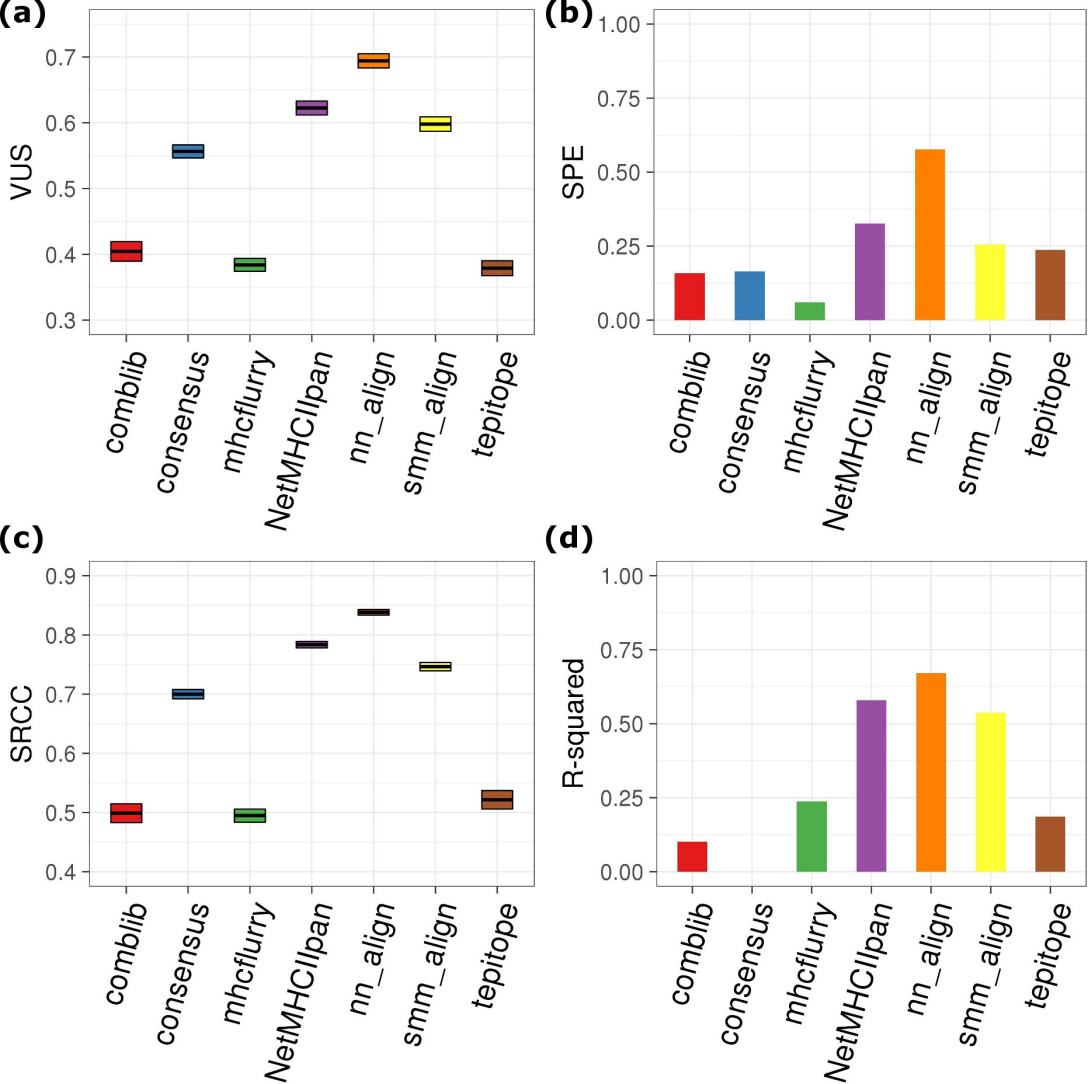
**Multiclass classification performance of MHC-II binding epitope prediction tools**: (a) VUS; (b) SPE; (c) SRCC; (d): R-squared of linear regression. The box plots of VUS and SRCC show values covering 95% confidence level.

As shown by Figs 5 and 6, ANN-based approach nn_align (NetMHCII2) exhibits a significant advantage of accuracy over other tools regarding the binding prediction of MHC class II epitopes. nn_align delivers an AUC value of 0.911 ± 0.004, with 80% TPr reached at the expense of ~ 13% FPr (Fig 5C). Further examination of ROC curves at high TPr range suggests that other two popular methods, including NetMHCIIpan and smm_align, have also been able to achieve < 20% FPr when reaching TPr of 80%. nn_align also achieves the highest VUS, SRCC and R-squared, with comparable performance to the best MHC class I prediction tools. The only exception in the metrics is SPE (0.671 for nn_align versus 0.836 for NetMHCpan3). This deficiency can be a result of the test set composition, as only 12.3% are strong MHC II binders (IC50 < 50 nM), while 38.8% are strong MHC I binders. In other words, MHC II binding prediction tools have a higher chance of falsely omitting strong binders, especially when the size of positive data available is small. Trailing nn_align on accuracy, NetMHCIIpan serves as a good alternative approach when the allele of interest is not yet trained by nn_align.

In the present study, we also employed the ANN framework of mhcflurry to train allele-specific MHC class II predictors using the same training set as of nn_align. For nn_align, smm_align, and NetMHCIIpan, this preference was determined *a priori* by Gibbs sampling of existing MHC II-binding sequences.[55] However, in contrast to these methods, class II mhcflurry did not consider separately the two 3-mer peptide flanking regions and the 9-mer binding core.[7] In other words, the binding core and the flanking regions bear the same weight as training input features. Instead, the 15-mer sequences were directly used as if all residues were assigned to binding region. The substandard performance of mhcflurry-MHC class II predictors (Fig 5, AUC = 0.740) indicates that this strategy has difficulty in capturing the correct sequence-binding affinity relationship. Evidently, the proper training to determine such relationship by ANN requires explicit consideration on the sequence matrix of flanking and binding core regions of peptides.

Despite the large sequence space of peptides bound to MHC proteins, the binding motif to a specific MHC protein is often characteristic. For example, the anchoring residues of MHC class I-binding 9-mer peptides exhibit heave amino acid preference at the 2^nd^ and 9^th^ position.[17,56] For this reason, even the simplistic PSSM approach can often predict the binding preference of peptides to MHC proteins with high accuracy, given a sufficient training set. However, in LR-based PSSM approaches such as smm and PickPocket, the contribution of each amino acid to overall MHC binding affinity is assumed to be independent.[13] Given evidence showing that pair-wise interactions between neighboring residues of MHC-binding peptides also influence the binding behavior[57], it is expected that ANN performs superiorly in terms of learning such complex features and handling regularization. This hypothesis is confirmed by our benchmarking on both MHC I and II binding prediction tools.

### Allele-specific prediction performance and training data size

Allele-specific binding prediction performance, measured by AUC for binary classification and by SRCC for affinity ranking, was consolidated as shown in S1 Fig. S2 Fig and S3 Fig show the ROC curves of individual MHC class I and II allele. The grey blocks on the heatmap suggest alleles that are not available for that particular method. At current state, allele-specific mhcflurry and pan-allele methods, including NetMHCpan2.8, NetMHCpan3, and PickPocket, accept the widest range of HLA alleles. For HLA type II, the training set of nn_align encompasses the most allele types.

The effect of training data size is examined by dividing HLA alleles into three groups, each with different sizes of MHC-binding peptide repertoire. For MHC class I, training data size has no significant impact on the performance of binary classification, as shown by lack of difference on heatmap across cyan, yellow and purple regime (S1 Fig). The same observation can be made based on boxplots of AUC (S1 Fig). Alleles with training set that has larger than 2500 peptides tend to achieve affinity ranking predictions better than alleles with binding repertoire smaller than 500 peptides. In contrast, larger training size for class II cases does not necessarily lead to better performance of neither binary classification (AUC) or affinity ranking (SRCC). This conclusion holds true in the case of allele-specific binding affinity threshold as well (S1 Fig).

This result suggests that while gaining more training data can potentially increase the accuracy of affinity ranking on specific alleles, significant improvement on the performance of identifying MHC-binding epitopes by binary classification is less likely expected. Our data shows that a dataset larger than 500 points may be sufficient enough as the training set for ML predictions. Depending on different cross-validation schemes employed, the size of allele-specific MHC-affinity array in pursuit needs to encompass both training and cross-validation sets, especially for the practical application of training new learning network for rare alleles.

### Absolute binding affinity prediction in strong binder space

**Fig 7:**
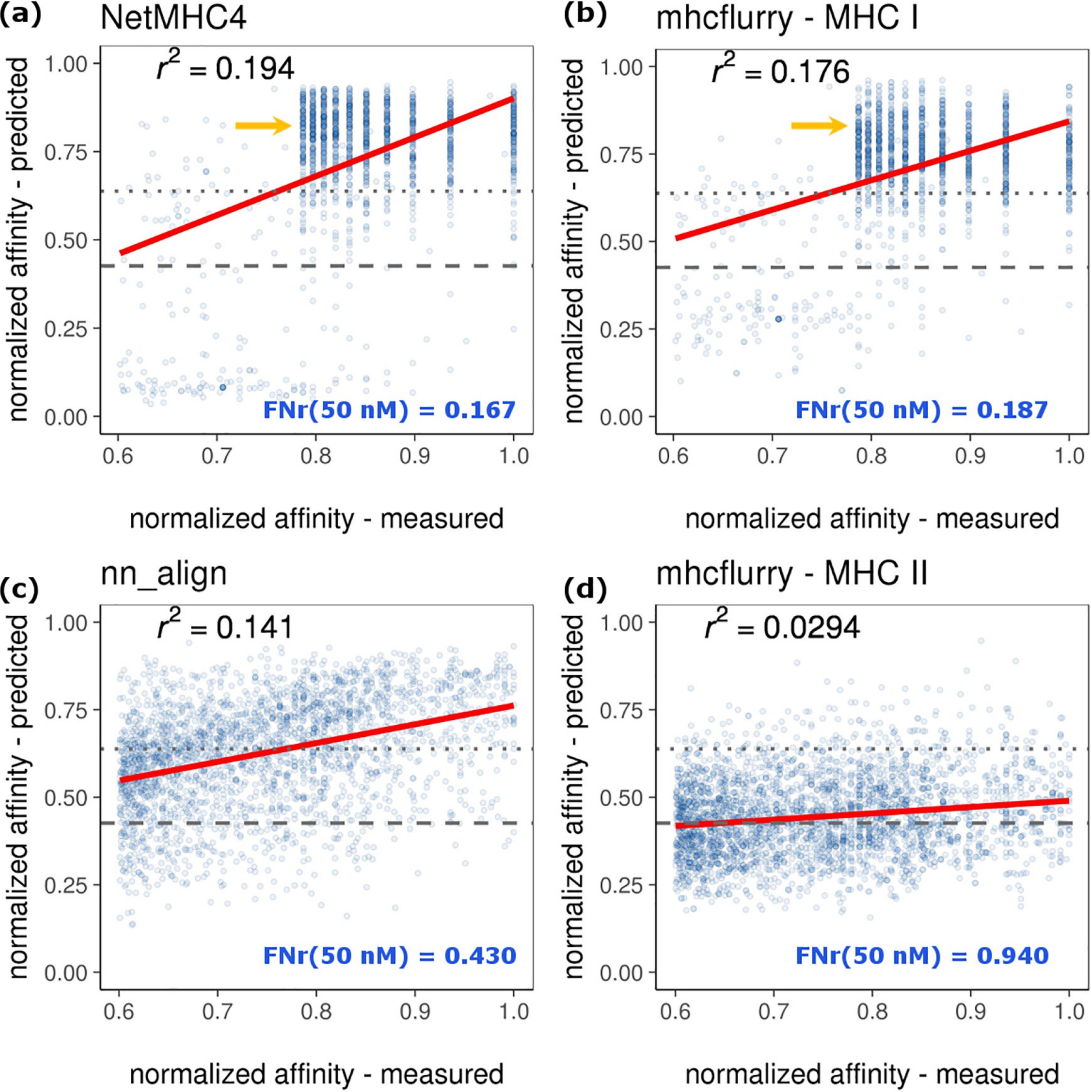
**Reliability of predicting absolute affinities of strong binding MHC Class I and II epitopes for (a) NetMHC4, (b) mhcflurry-class I, (c) nn_align, and (d) mhcflurry-class II.** Measurement and prediction values were represented as *1-log10(IC50)/log10(50000 nM)* and were light-colored based on 2-D data density. Grey dotted lines mark 50 nM threshold (y = 0.638) and grey dashed lines mark 500 nM (y = 0.426) threshold. Red lines show the linear regression of the data. *FNr(50 nM)* indicates the false negative rate of classifying strong binders.

While ANN-based approaches have given satisfying performance on the classification of MHC binders, the prediction on accurate absolute binding affinity has been less scrutinized. As already shown by Figs 4D and 6D, the linear correlation between predicted and measured IC50 values does not adequately support the prediction as a good indicator for absolute binding affinity of untrained sequences. In practical applications, a strong binder threshold of IC50 = 50 nM is often used to further identify the most potent epitopes; therefore we plotted and fitted the regression between predicted and measured IC50 at the stronger binder regime for MHC class I and II test data (Fig 7). As suggested by the decreased R-squared, the correlation is deteriorated than the whole IC50 range (Figs 4D and 6D). A fair amount of points which represent measured strong MHC-affinity were incorrectly predicted to be non-binders or weak binders (Fig 7, points below grey dashed and dotted lines). The FNr of strong binder classification, as shown in Fig 7, suggest the high risk of false filtering when applying 50 nM as cutoff for peptide binders. Furthermore, MHC I binding predictors actually generated diverge predictions for peptides that have highly similar measured affinity (Fig 7, yellow arrows.)

Previous studies have reported that the sequences identified from MHC-peptide binding predictions often resulted in a sparse immunogenic space.[31,42] Clearly, the false negative rate and the weak correlation between measured and predicted affinities of these predictors can be key sources of errors. While these predictors can identify the sequence pool of strong binders relatively well by classification, prioritizing antigen candidates by ranking the predicted MHC binding affinities may not be appropriate. One caveat to recognize is the sensitivity or resolution of experimental binding assays in measuring high affinity zone, which leads to the inaccuracy in training the predictors. Therefore, extra caution is required when applying the predictors, such as applying the predicted relative ranking score instead of the affinity for minimizing the discrepancy between predicted and experimental absolute affinity values.

### MHC-eluted peptides prediction capacity

While MHC-binding is postulated to be the most important step for antigen processing and presentation, subsequent steps involving proteasome cleavage of proteins and transporter-associated processing (TAP) contribute together to determine the final peptidome displayed on APC surface. Methods such as NetChop[58] and NetCTL[59] were devised to train ANN to predict such events. Due to the smaller amount of high-quality data available for training, however, these predictions can hardly achieve the same level of classification reliability compared to binding prediction tools such as NetMHC. Thus in practical applications of antigen identification, often MHC-binding prediction is relied solely upon. Recent efforts have emerged for developing the reliable prediction of naturally APC-presented peptides, by training ANN on a set of MHC-eluted peptide sequences that were obtained from high-resolution and high-throughput liquid chromatography-mass spectrometry (LC-MS) experiments.[17,43,54,60,61] Here we assessed the ability of currently benchmarked tools to identify MHC-eluted peptides correctly. The test sets include three MS-derived datasets from recent studies (Methods).[62]

**Fig 8:**
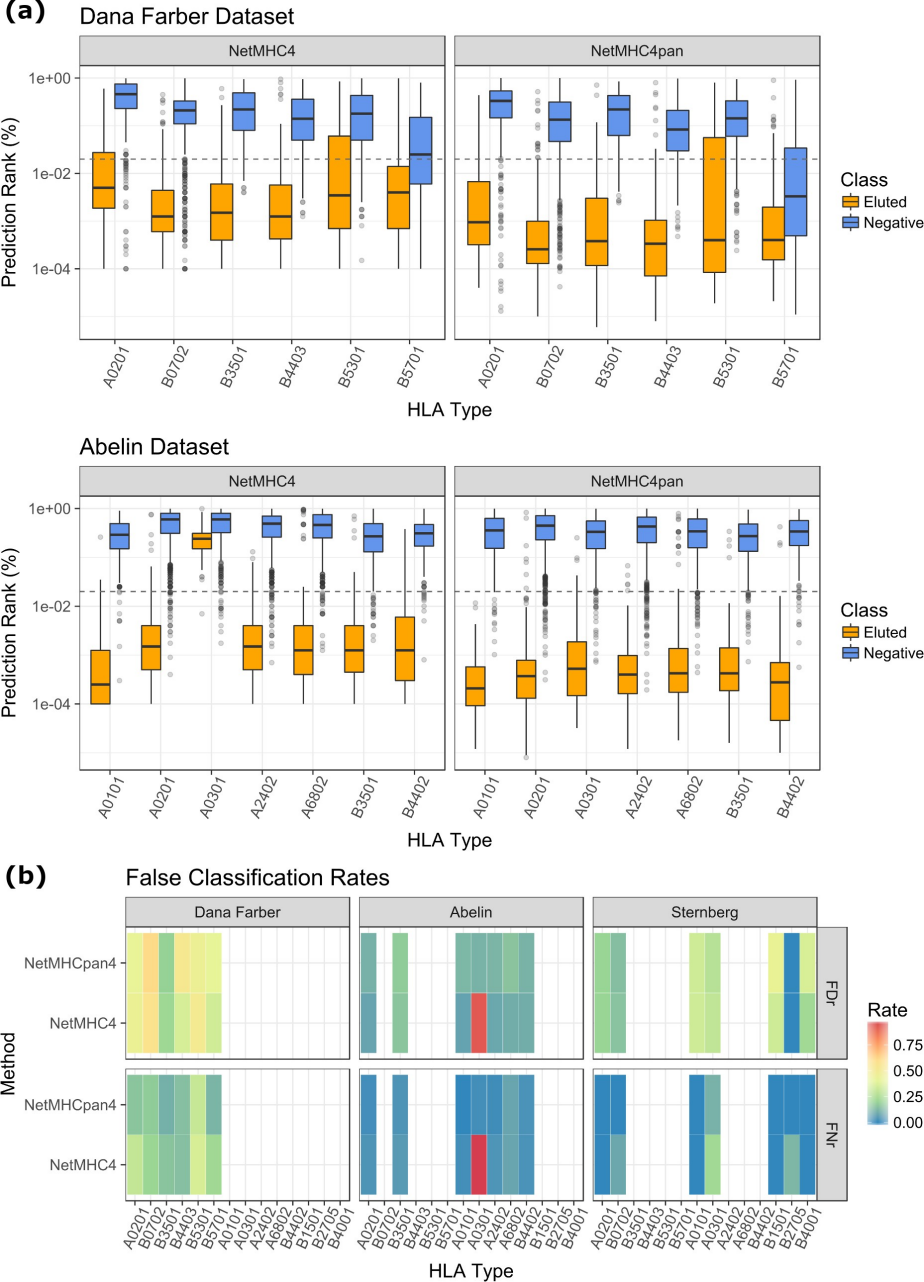
Assessing the reliability of binding prediction methods for the identification of naturally processed MHC-epitopes. (a) Box plots showing the quartile distribution of binding affinity rankings as predicted by NetMHC4 and NetMHCpan4 for MHC-eluted and non-eluted peptides. Grey dashed line indicates predicted binder ranking of top 2%. (b) FDr and FNr values calculated based on top 2% percentile rank cutoff on three MS-derived datasets visualized as heatmap. Similar plots of predicted scores are shown in S4 Fig.

Across the three datasets, we obtained varied accuracy for NetMHC4, NetMHCpan4, and MixMHCpred methods (Figs 8 and S4). We considered the primary cutoff using ranking score, in that the binding affinity threshold (IC50) should not be applicable in the scenario of predicting elution probability. In addition, similar to the heterogeneous nature of binding repertoire, the MHC-eluted repertoire will mostly likely vary in size and affect the allele-specific cutoff. Overall, NetMHCpan4 achieves better accuracy compared to NetMHC4, which is expected as it considered elution data in addition to binding affinity data for training. Due to the fact that the negative class in Abelin and Sternberg testing sets are synthetic peptides randomly drawn from MS peptidome database, their binding affinities are also very low, leading to high accuracy of both NetMHCpan4 as well as NetMHC4 (Fig 8A and S4 Fig). In contrast, the negative class in Dana Farber testing set is composed of predicted MHC binders. In this case, NetMHCpan4 overall achieves lower false prediction rates than NetMHC4. Similar trend can also be observed when using predicted binding affinity/elution score as cutoff (S4 Fig). MixMHCpred also has improved accuracy than NetMHC4 on the three testing data when comparing prediction score using a putative cut-point of 0.5. Its low false prediction rates are also close to NetMHCpan4, with the improvement of zero false discovery rate (FDr) on B4403 allele and zero false negative rate (FNr) on A0301 allele. When considering all the prediction tools on Dana Farber data, NetMHCpan4 performs the best across six HLA types (S5 Fig), which is likely due to that the dataset has already been included in the training set.

One potential caveat of binding affinity predictors for tasking eluted peptide prediction is the relative high FDr, in that MHC-eluted peptides identified by MS experiments were often observed to have much smaller repertoire size than MHC-binding peptides. This caveat is also demonstrated here, with FDr reaching as high as 54% (Fig 8B, HLA-B0702 in Dana Farber testing set). While FNr is generally lower than FDr, significant allelic variability was also observed, with FNr reaching 30% for certain HLA types. Considering that the repertoire size of actual MHC-presented epitopes is often limited, the misclassification of 3 out 10 epitopes can be critical during experimental validations.

These observations demonstrate the variability of MHC-binding predictors when used for classifying antigen presentation, as shown by the non-trivial FPr and FNr. We note that, due to the inherit difference in repertoire sequences of MHC-binding and MHC-presenting peptides, it is reasonable for prediction methods trained on one type of data to perform sub-optimal on another type. In general, ML-based MHC-binding prediction tools are capable of achieving decent AUC values for classifying eluted versus non-eluted antigen-processed peptides, especially with the recent development of NetMHCpan4 and MixMHCpred. Nevertheless, the FNr and FDr should be taken into rigorous account in a cancer vaccine prediction pipeline.

### MHC I binding prediction by peptide-protein docking

**Fig 9:**
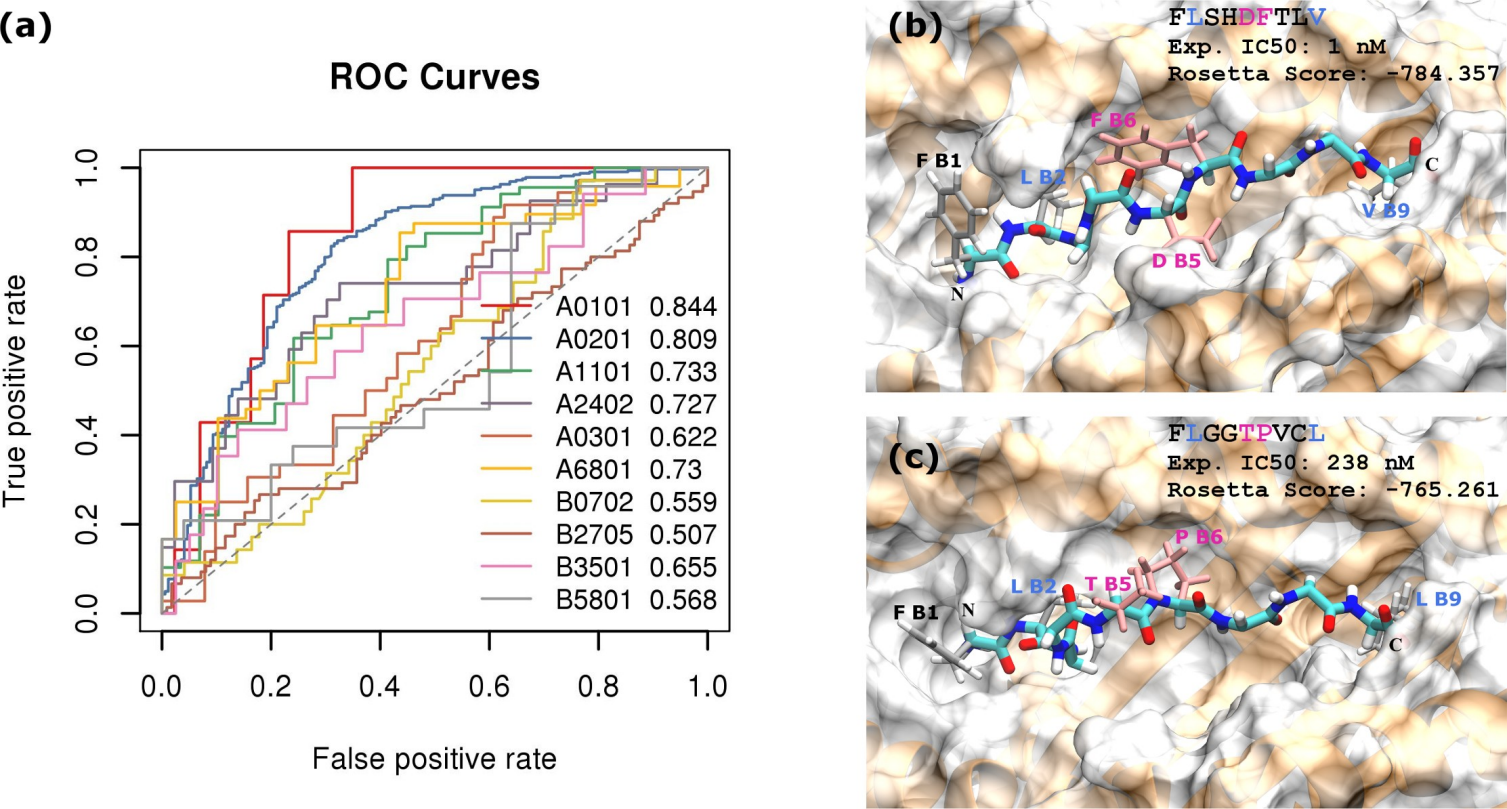
Prediction of MHC class I epitopes by FlexPepDock. (a) ROC curves and AUC values (shown after allele legend) generated based on reweighted binding energy scores reported by FlexPepDock. Peptides were labeled as positive or negative class by the IC50 = 500 nM cutoff. True positive and false positive were then calculated by correlating with FlexPepDock reweighted score. (b) and (c) Lowest energy conformations of 9-mers FLGGTPVCL and FLSHDFTLV to HLA-A0201 protein. MHC proteins are shown by orange ribbon and white surface; peptide backbones are shown in cyan; MHC-binding residues are shown in silver; potential T cell receptor contacting residues are shown in pink.

We applied structure-based peptide-protein docking protocol FlexPepDock to model the binding of 9-mer peptides to MHC class I proteins. Fig 9A shows the ROC curves and AUC benchmarking classification by Rosetta Score. The accuracy of the binary classification is at the lower spectrum end when put in line with ML-based approaches. In order to achieve a TPr of 80%, the Rosetta predictor commits about 50% FPr or higher for most alleles. Consistent with previous modeling study, while FlexPepDock displays as an operating predictor for differentiating MHC binders of certain alleles, it suffers from the insufficiency of backbone conformational sampling. In the current FlexPepDock protocol, although backbone structure was optimized before side chain refinement, the flexibility of MHC-binding epitope backbone made searching of the vast conformational space exhaustively challenging. Thus, the accuracy of final optimized model still largely depends on the template crystal structure chosen.[32]

However, the peptide-MHC complex structure rendered by FlexPepDock still provides useful information at 3-D level. We extracted the conformation and energy of two HLA-A0201 binding epitopes from modeling results and analyzed the peptide-MHC binding pocket (Fig 9B and 9C). Rosetta FlexPepDock correctly ranked FLSHDFTLV (FLS) (exp. IC50 = 1 nM, Rosetta score = -784.357) above FLGGTPVCL (FLG) (exp. IC50 = 238 nM, Rosetta score = -765.261) as stronger binder. The binding conformations for both peptides are stabilized by the fitting of leucine (L, B2 and B9) or valine (V, B9) side chains into the hydrophobic pocket on MHC surface (Fig 9B and 9C, blue labels). These two amino acids were confirmed to be the primary anchoring residues for epitope binding to A0201.[17,32] On the other hand, we found that stronger binder FLS may not necessarily be an ideal candidate for eliciting immunogenicity, in that its 5^th^ and 6^th^ residues, aspartic acid and phenylalanine respectively, actually pose side chains towards the MHC pocket as well (Fig 9B). In contrast, slightly weaker binder FLG poses 5^th^ and 6^th^ residues threonine and proline preferably outwards, making their contact with T-cell receptor more energetic favorable (Fig 9C).[29,63,64] In this case, positive epitopes from binding affinity-based prediction may not entirely conform to antigens that promote strong T-cell receptor binding and immunogenicity. Structural modeling-based approaches such as Rosetta FlexPepDock, hence, provide an alternative avenue to complement the analysis pipelines towards identifying vaccine candidates.

## Discussion

### Improving the prediction accuracy of absolute binding affinity

Our data suggests that while current MHC-binding predictors achieve high accuracy on classifying MHC-binders and non-binders, their performance on delivering precise binding affinities are inferior. This problem is almost intrinsic to ML-based approaches: the effect of the most dominant features on the weight matrix is penalized by regularization intentionally to achieve better generalization on those blind test data with less dominant features.[65] While this setting is designed to solve the classification problem, it limits the extent of recovering the absolute binding affinity by regression prediction. One source of the inaccuracy roots in the loss of sensitivity of experimental assays at either very high or low binding affinity regimes. Another related error may come from imbalance of training data across different affinity tiers. As a consequence, epitope candidates for subsequent experimental validation selected by ranking the predicted binding affinities may not necessary reflect the *in vivo* affinity values.

Molecular modeling-based technique can calculate peptide-MHC binding free energy with high fidelity to experimental value.[66] Due to the limitation on computational cost, this solution would be applied only as a downstream, detailed analysis for structural interaction between peptides and MHC.[30] Imprecise affinity prediction can also lead to inferior classification performance for certain alleles, in that arbitrary affinity thresholds are often used in practice (i.e., 500 nM for binders and 50 nM for strong binders). Such thresholds were shown to underestimate MHC-binding peptide repertoire for rare HLA alleles.[51] To alleviate this limitation, percentage rank, instead of binding affinity, has been introduced to rank epitope candidates. Increasing the prediction accuracy of absolute binding affinity for ML-based approaches remains to be a major direction of improvement.

### Predicting different peptide length

In the current study, it is demonstrated that majority of the methods are able to attain comparable prediction performance between MHC class I 9-mer and 10-mer ligands. This observation is in concordance with previous testing on these methods, which employ strategies of designing gap and insertion[18], or different training matrices for mapping different length[17]. We note that expanding the benchmarking to other length of MHC-ligands or immunogenic peptides is of significant relevance for understanding length preference of T-cell epitopes, which will be considered in our future work.

### Combining synthetic with naturally processed MHC-peptidome

The MHC-peptidome repertoire used to train binding affinity prediction tools has included a substantial amount of artificially synthesized peptides. While this inclusion has largely enhanced the sequence space of potential MHC-binders, it also generated bias in training set. The predictions from these tools lead to a skewed population of binding motifs that do not necessarily consider APC endogenous processing and TAP. As seen from our benchmarking of IEDB tools, the precision of the predictors in identifying naturally processed MHC-binders is suboptimal compared to predicting binding affinity.

Mass spectrometry (MS) of “pull-down” experiment, taking advantage of MHC-specific antibodies, yields a relatively unbiased sampling of naturally processed endogenous peptides, and can correct the training set bias introduced by synthetic sequences. Moreover, it has been reported that a considerable amount of peptides eluted from MHC molecules are in fact proteasome-spliced sequences.[27] MS-based naturally processed MHC-peptidome is expected to facilitate the discovery of such non-canonical MHC-binding motifs as well. Increasing number of studies have focused on generating such MS-based MHC-peptidome dataset[43,67,68], while a large fraction of the results are scattered and yet to be included as new training set. Incorporating large-scale MS-based MHC-peptidome data with existing binding affinity and antigen-processing predictors[17,42,43,54], as demonstrated by our benchmarking of NetMHCpan4 and MixMHCpred versus NetMHC4, is capable to improve the identification accuracy of naturally processed epitopes.

Overall, the integration of multiple data sources in the antigen presentation and T-cell interaction pathways should be considered for efficient identification of vaccine candidates. For example, current MS-based MHC-peptidome data still lack good concordance with peptides predicted to be strong MHC binders. Our benchmarking indicates that binding affinity predictors have varied accuracies on MS-identified peptides, suggesting potential bias can be introduced when analyzing original MS data with predicted affinity filtering. For example, the Sternberg dataset was filtered by NetMHC4 prediction to reduce non-binding negatives and therefore is subject to bias when benchmarked on this predictor. A recent study has alleviated such issue with MHC-peptidome deconvolution[17], which also provides future direction to assess the soundness of different predictors on unbiased MHC-peptidome testing sets. In addition to curating high-quality MHC-peptidome data, considerable upgrading of prediction power can potentially be gained by combining feature descriptors related to MS data, such as protein abundance, to fully realize the advantage of ANN approach[42]. This strategy offers a path towards a deep understanding of antigen presentation process.

### Evolving from MHC-binding prediction to T cell epitope prediction

The ultimately goal of MHC-binding and other antigen presentation predictions is to identify peptides for eliciting adaptive immune response. In particular, the success of cancer immunotherapy has opened a venue for applying personalized cancer vaccine based on individual’s HLA allele types and tumor profile. Tumor-associated antigens or neoantigens are prominent candidates for cancer vaccine. *In silico* prediction methods are expected to prioritize antigens based on their potentials to elicit T cell responses, yet only a small fraction of predicted candidates turned up to be immunogenic in many case studies.[26] One reason is the lack of database reporting the relationship between epitope sequences and the associated T cell immunogenicity. Current high-throughput approach using tetramer staining of tumor infiltrating lymphocytes[69] has only produced a limited amount of affinity matrix as training set. As shown in this paper, alternative approach using structure-based modeling may be used to predict TCR-peptide-MHC interaction without prior knowledge. However, compared to the interactions between peptide epitope and MHC, the recognition of TCR to peptide-MHC is much more complex, due to the lack of well-defined binding groove at TCR protein surface. This complexity raises a tremendous amount of computational burden in practice. Despite these difficulties, we argue that the current prediction algorithms are necessitated to evolve towards T cell epitope prediction, in order to transform personalized cancer vaccine and biomarker development into practices that are approachable beyond research laboratory.

### Summary

In this study, we performed a systematic and quantitative benchmarking of popular MHC class I and II-binding prediction methods by using a comprehensive evaluation metrics. We also developed mhcflurry into a pan-HLA prediction approach to facilitate its application on HLA types with insufficient training data. For MHC class I, mhcflurry (AUC = 0.911) and consensus (AUC = 0.968) was demonstrated to be the best binary classifier on 9-mers and 10-mers, respectively. mhcflurry also achieved the best for multi-class classification and relative affinity ranking. Pan-HLA version of mhcflurry have the best accuracy in identifying strong MHC I-binders (IC50 < 50 nM). For MHC class II, nn_align/NetMHCII2 (AUC = 0.911) constantly outperformed other tools on all evaluation standards. The current binding prediction tools have achieved tremendous accuracy with respect to categorical classification of strong MHC-binders from the test set, demonstrating the advances made by large-scale synthetic peptide-MHC binding dataset and state-of-the-art ML approaches. On the other hand, important lessons have also been learnt as to the deficiency of current algorithms in predicting absolute binding affinity. With respect to identifying naturally processed MHC-peptidome using predicted ranks, variability of accuracy has been observed between different testing data, while the newly developed tool NetMHCpan4 displayed good performance compared to conventional binding affinity predictors. The contrast between results on MHC-binding and MHC-elution epitopes presents a new view of best practice in T-cell epitope prediction. In addition, we conducted extensive benchmarking of structure-based peptide-MHC binding prediction by Rosetta FlexPepDock, demonstrating the usage and weakness of structural modeling for antigen identification. Our benchmarking results provided an overall guideline regarding the predictive capacity of MHC-binding predictors and the potential directions of improvement for their applications in personalized cancer vaccine design and development.

## Supporting information

S1 Table List of binding affinity data volume for each HLA Class I and II alleles benchmarked in this study. (PDF)

S2 Table List of PDB structures used as initial template for FlexPepDock modeling. (PDF)

S1 Fig The size of training sets has a minor impact on the performance of MHC I and II binding predictions. (PDF)

S2 Fig ROC curves of HLA Class I binding epitope prediction tools for each individual allele. (PDF)

S3 Fig ROC curves of HLA Class II binding epitope prediction tools for each individual allele. (PDF)

S4 Fig Additional data on the accuracy of NetMHC4, NetMHCpan4, and MixMHCpred on predicting three MS-derived elution datasets. (PDF)

S5 Fig ROC curves and corresponding AUC values for predicting eluted *vs*. non-eluted MS-confirmed peptides in Dana Farber dataset. (PDF)

S6 Fig ANN layer architecture of mhcflurry_pan. (PDF)

S1 Dataset Testing 9-mer and 10-mer peptide sequences used for benchmarking HLA Class I prediction methods. (ZIP)

S2 Dataset Testing 15-mer peptide sequences used for benchmarking HLA Class II prediction methods. (ZIP)
