# Supplementary material for: Systematically benchmarking peptide-MHC binding predictors: From synthetic to naturally processed epitopes
Source: PLoS Comput Biol. 2018 Nov 8;14(11):e1006457. doi: 10.1371/journal.pcbi.1006457 (PMC6224037; doi:10.1371/journal.pcbi.1006457)
Supplement: S1 Table — (PDF) [file pcbi.1006457.s001.pdf]

Table S1. List of binding affinity data volume for each HLA Class I and II allele (9-mer and 15-mer respectively) benchmarked in this study. The test data were derived by subtracting the most recent version of training datasets included by the prediction tools from all quantitative MHC-ligand binding assay data curated in IEDB database (as of Oct. 2016). For HLA class II alleles, grey font entries represent alleles that have yet to be trained for IEDB tools and thus are excluded from the current benchmarking.

HLA Class I Allele	**# of test data**	% of strong binder (*IC50 < 50 nM*)	% of weak binder (*50 nM < IC50 < 500 nM*)	% of non-binder (*IC50 > 500 nM*)	# of training data included by prediction tools
A*01:01	**51**	9.8	3.9	86.3	1409
A*02:01	**426**	42.7	9.6	47.7	5441
A*02:02	**152**	81.6	13.8	4.6	1980
A*02:03	**115**	80.9	0	19.1	2571
A*02:05	**27**	0	81.5	18.5	34
A*02:06	**88**	64.8	29.5	5.7	2680
A*03:01	**83**	25.3	18.1	56.6	2658
A*11:01	**93**	61.3	8.6	30.1	2431
A*24:02	**70**	25.7	15.7	58.6	1421
A*29:02	**47**	38.3	0	61.7	1066
A*30:01	**77**	96.1	1.3	2.6	1180
A*30:02	**93**	43	16.1	40.9	846
A*31:01	**74**	48.6	0	51.4	2113
A*32:01	**37**	97.3	0	2.7	645
A*33:01	**57**	12.3	0	87.7	1299
A*68:01	**85**	43.5	11.8	44.7	1631
A*68:02	**105**	40	0	60	2085
B*07:02	**108**	14.8	17.6	67.6	1792
B*08:01	**38**	76.3	2.6	21.1	1291
B*15:01	**33**	97	0	3	1951
B*15:03	**72**	100	0	0	448
B*27:04	**30**	0	0	100	1
B*27:05	**188**	1.1	37.8	61.2	856
B*27:06	**26**	0	3.8	96.2	2
B*35:01	**96**	14.6	3.1	82.3	1265
B*38:01	**177**	28.8	41.2	29.9	217
B*44:03	**72**	2.8	4.2	93.1	494
B*51:01	**36**	11.1	0	88.9	720
B*53:01	**113**	13.3	0	86.7	638
B*54:01	**44**	27.3	18.2	54.5	434
B*57:01	**65**	12.3	13.8	73.8	673
B*58:01	**49**	28.6	20.4	51	986

Public

HLA Class II Allele	**# of test data**	% of strong binder (*IC50 < 50 nM*)	% of weak binder (*50 nM < IC50 < 1000 nM*)	% of non-binder (*IC50 > 1000 nM*)	# of training data included by prediction tools
DPA1*01:03/DPB1*02:01	**354**	5.6	16.1	78.2	1772
DPA1*01:03/DPB1*03:01	**164**	17.7	32.3	50	0
DPA1*01:03/DPB1*04:01	**807**	5	20.6	74.5	0
DPA1*01:03/DPB1*04:02	**38**	0	26.3	73.7	0
DPA1*02:01/DPB1*01:01	**496**	4.2	25.4	70.4	1832
DPA1*02:01/DPB1*05:01	**556**	4.1	17.8	78.1	1589
DPA1*03:01/DPB1*04:02	**512**	5.1	18.9	76	1793
DPB1*01:01	**100**	10	46	44	0
DPB1*02:01	**164**	12.8	44.5	42.7	0
DPB1*03:01	**99**	24.2	37.4	38.4	0
DPB1*04:01	**105**	4.8	30.5	64.8	0
DPB1*04:02	**99**	8.1	28.3	63.6	0
DPB1*05:01	**100**	17	41	42	0
DPB1*14:01	**164**	7.9	22.6	69.5	0
DPB1*20:01	**164**	7.9	26.2	65.9	0
DQA1*01:01/DQB1*05:01	**606**	3.3	11.7	85	1464
DQA1*01:02/DQB1*05:02	**164**	4.9	29.3	65.9	0
DQA1*01:02/DQB1*06:02	**477**	8.6	33.3	58.1	1776
DQA1*01:04/DQB1*05:03	**164**	2.4	26.2	71.3	0
DQA1*02:01/DQB1*02:02	**164**	1.8	21.3	76.8	0
DQA1*03:01/DQB1*03:01	**49**	12.2	26.5	61.2	0
DQA1*03:01/DQB1*03:02	**514**	0.4	17.9	81.7	1751
DQA1*04:01/DQB1*04:02	**518**	2.7	20.5	76.8	1711
DQA1*05:01/DQB1*02:01	**535**	1.5	19.8	78.7	1680
DQA1*05:01/DQB1*03:01	**486**	27.2	36.2	36.6	1881
DQB1*02:01	**100**	2	44	54	0
DQB1*02:02	**94**	1.1	18.1	80.9	0
DQB1*03:01	**133**	21.8	32.3	45.9	0
DQB1*03:02	**126**	1.6	32.5	65.9	0
DQB1*04:02	**99**	7.1	45.5	47.5	0
DQB1*05:01	**93**	3.2	36.6	60.2	0
DQB1*05:02	**93**	1.1	28	71	0
DQB1*05:03	**94**	0	27.7	72.3	0
DQB1*06:02	**98**	11.2	45.9	42.9	0
DRB1*01:01	**875**	30.3	35.9	33.8	13204
DRB1*03:01	**863**	12.3	18.5	69.2	3685
DRB1*04:01	**881**	20.2	34.5	45.3	4195
DRB1*04:04	**369**	14.9	38.5	46.6	1856

Public

DRB1*04:05	**692**	7.1	30.5	62.4	3507
DRB1*07:01	**847**	22.9	33.5	43.6	4046
DRB1*08:01	**14**	7.1	14.3	78.6	0
DRB1*08:02	**821**	20.6	31.2	48.2	3355
DRB1*09:01	**647**	14.7	34.9	50.4	3613
DRB1*10:01	**165**	53.3	41.8	4.8	0
DRB1*11:01	**802**	20.9	26.4	52.6	4062
DRB1*12:01	**813**	3.2	28.5	68.3	0
DRB1*13:02	**866**	23	26.4	50.6	3405
DRB1*15:01	**906**	19.2	32.7	48.1	3981
DRB1*16:02	**123**	13	64.2	22.8	0
DRB3*01:01	**635**	3.9	15.4	80.6	3102
DRB3*02:02	**811**	6.2	16.3	77.6	0
DRB4*01:01	**721**	9.6	27.6	62.8	3445
DRB5*01:01	**712**	14	34	52	4058

P Public
