# Supplementary material for: Systematically benchmarking peptide-MHC binding predictors: From synthetic to naturally processed epitopes
Source: PLoS Comput Biol. 2018 Nov 8;14(11):e1006457. doi: 10.1371/journal.pcbi.1006457 (PMC6224037; doi:10.1371/journal.pcbi.1006457)
Supplement: S2 Table — (PDF) [file pcbi.1006457.s002.pdf]

Table S2. List of PDB structures used as initial template for FlexPepDock modeling.

HLA allele	PDB ID	# of test data
A0101	3bo8	51
A0201	3mrg	426
A0301	2xpg	83
A1101	1x7q	93
A2402	2bck	70
A6801	4hwz	85
B0702	5eo0	108
B2705	2a83	188
B3501	2cik	96
B5801	5ind	49

Public
