# Supplementary material for: Systematically benchmarking peptide-MHC binding predictors: From synthetic to naturally processed epitopes
Source: PLoS Comput Biol. 2018 Nov 8;14(11):e1006457. doi: 10.1371/journal.pcbi.1006457 (PMC6224037; doi:10.1371/journal.pcbi.1006457)
Supplement: S1 Fig — (PDF) [file pcbi.1006457.s003.pdf]

Fig S1. **The size of training sets has a minor impact on the performance of MHC I and II binding predictions.** (a) The size of training set included in IEDB tools defines three allele groups for HLA type I and II respectively, as colored by cyan, yellow, and purple in the side color bar. AUC and SRCC values are indicated by red-white-blue colored key. Grey blocks indicate alleles not available for specific tools. For allele B2704 and B1503, only binder or non-binder data is available so AUC is not shown. (b) MHC class I prediction AUC using default cutoff of 500 nM or using allele-specific cutoff as defined in Paul et al. (2013). Classification of HLA type by training data size is the same as of (a).

(a)

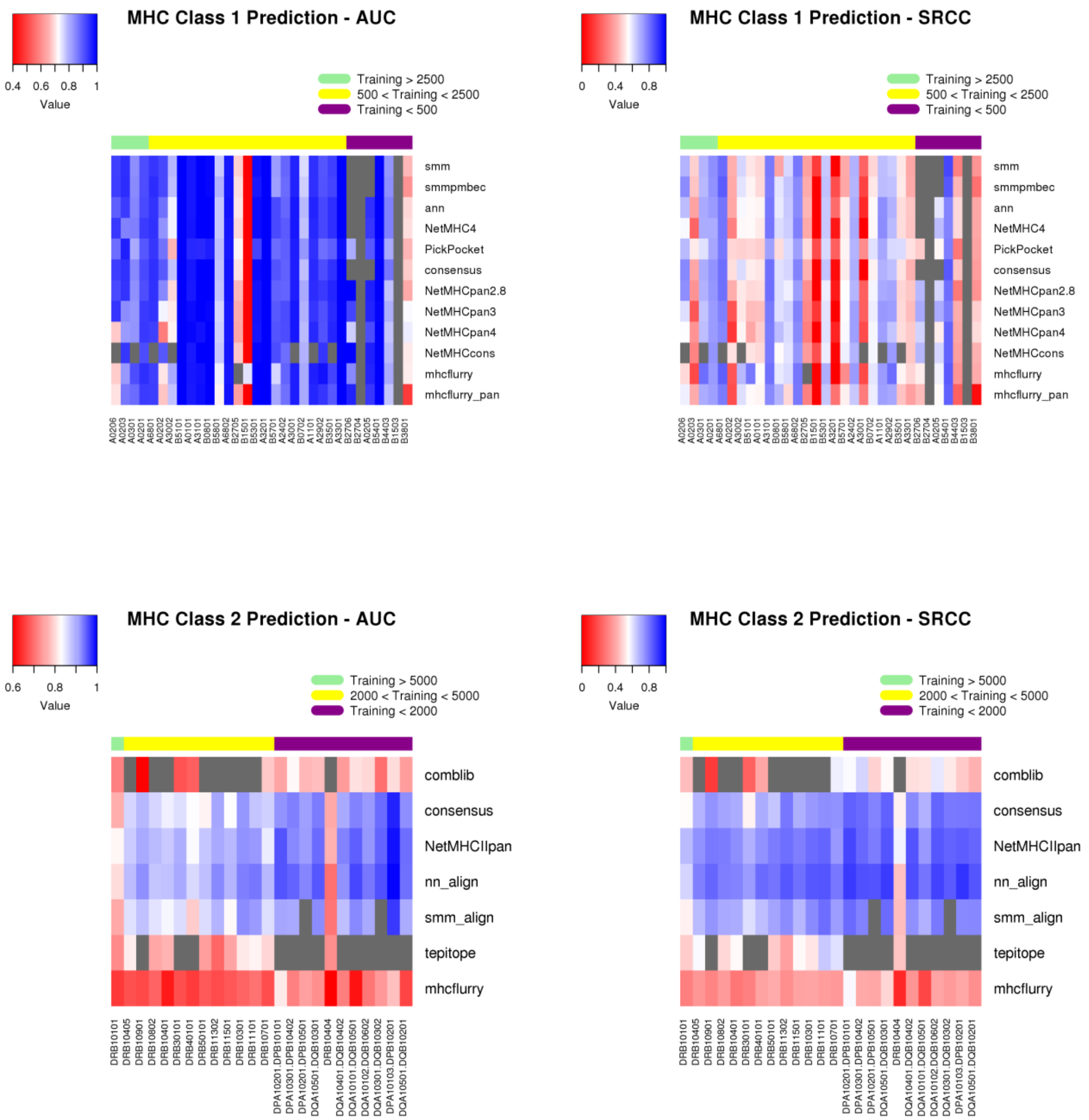

Public

(b)

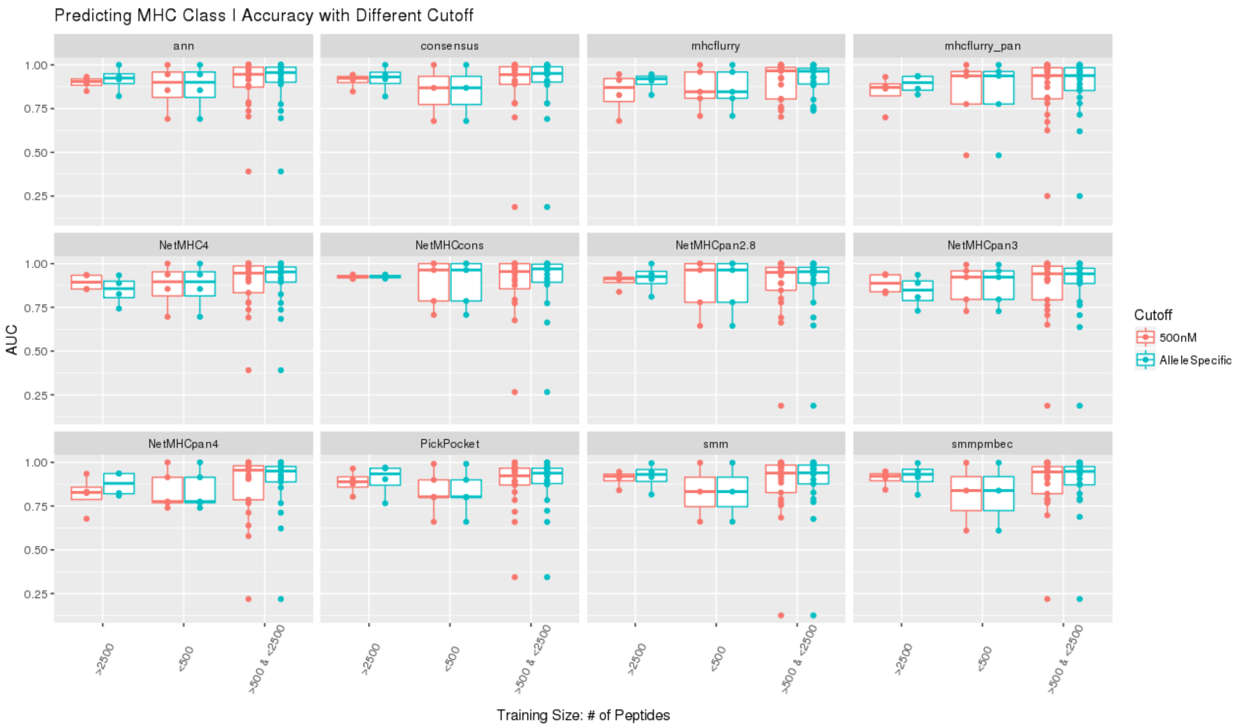
Predicting MHC Class I Accuracy with Different Cutoff
ann
consensus
mhcflurry
mhcflurry_pan
NetMHC4
NetMHCcons
NetMHCpan2.8
NetMHCpan3
NetMHCpan4
PickPocket
smm
smmpmbec
AUC
1.00
0.75
0.50
0.25
>2500
<500
>500 & <2500
Training Size: # of Peptides
Cutoff
500nM
AlleleSpecific
