# Supplementary material for: Systematically benchmarking peptide-MHC binding predictors: From synthetic to naturally processed epitopes
Source: PLoS Comput Biol. 2018 Nov 8;14(11):e1006457. doi: 10.1371/journal.pcbi.1006457 (PMC6224037; doi:10.1371/journal.pcbi.1006457)
Supplement: S2 Fig — (PDF) [file pcbi.1006457.s004.pdf]

Fig S2. **ROC curves of HLA Class I binding epitope prediction tools for each individual allele.**

smm

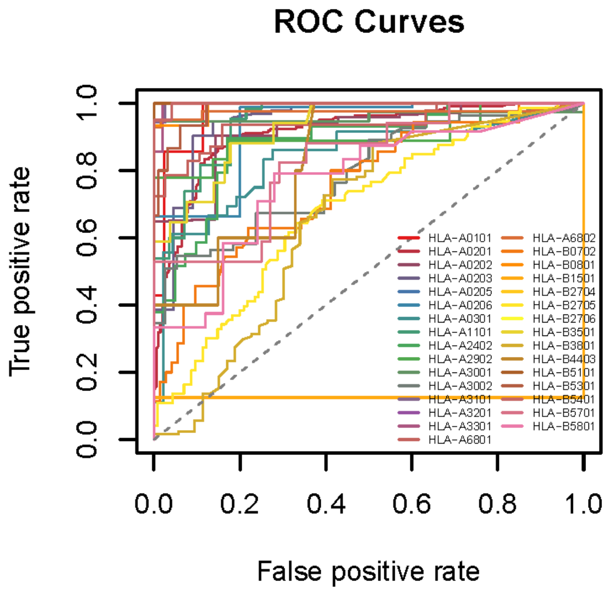

smmpmbec

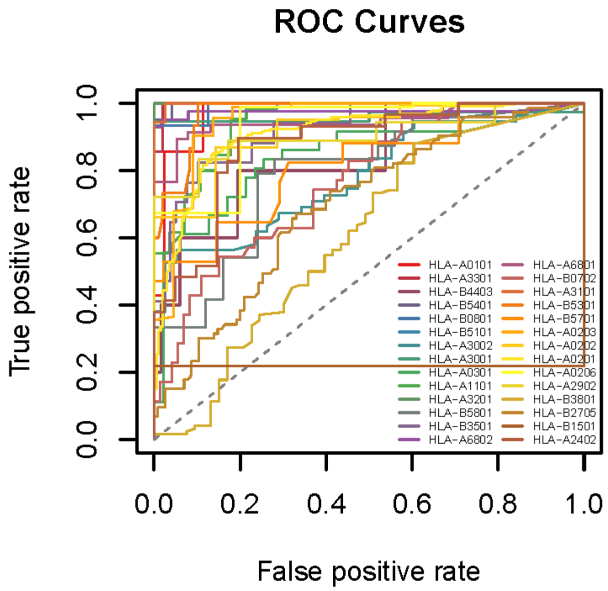

ann (NetMHC3.4)

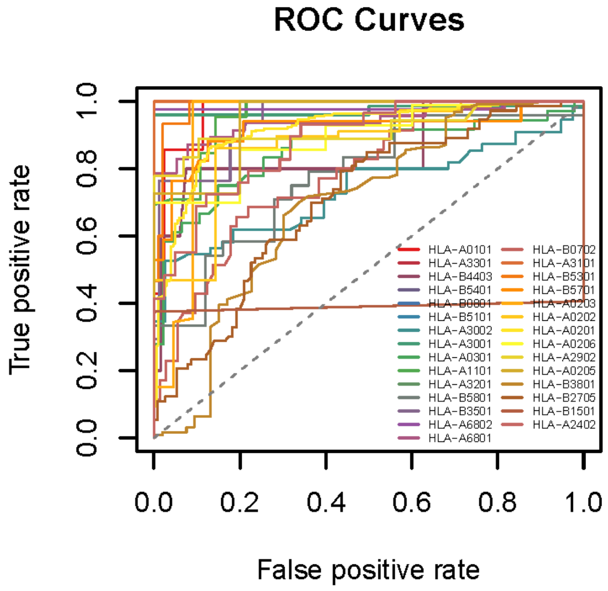

NetMHC4

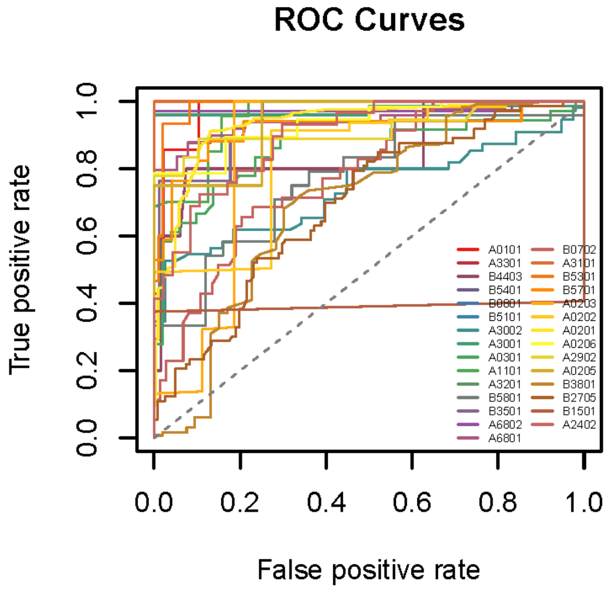

Public

NetMHCpan2.8

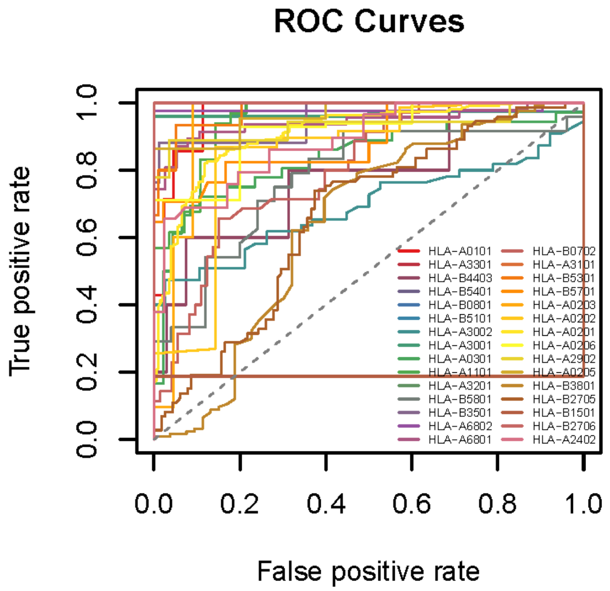

NetMHCpan3

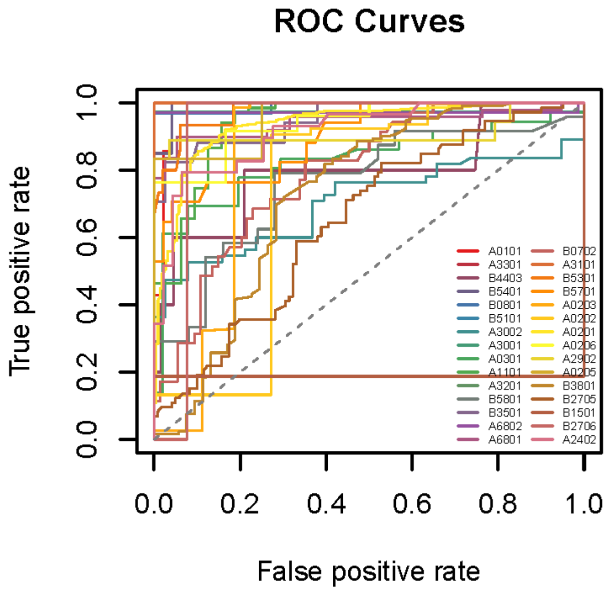

PickPocket

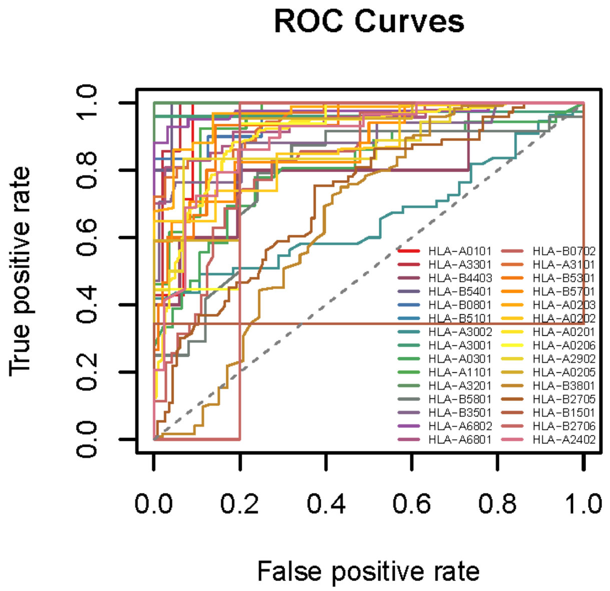

consensus

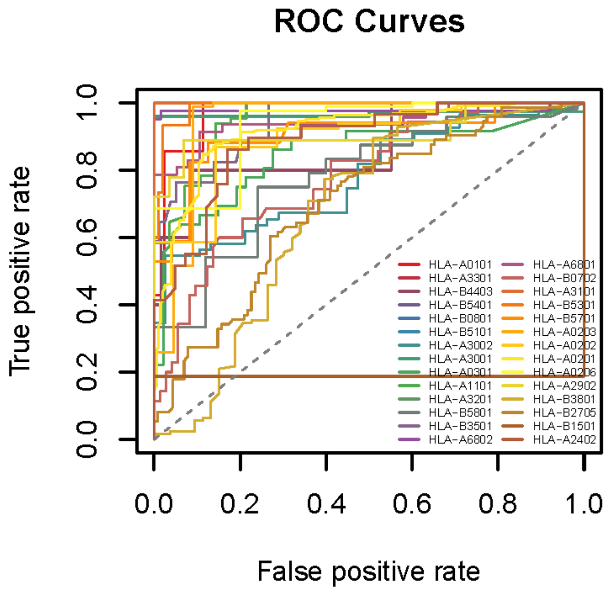

Public

NetMHCcons

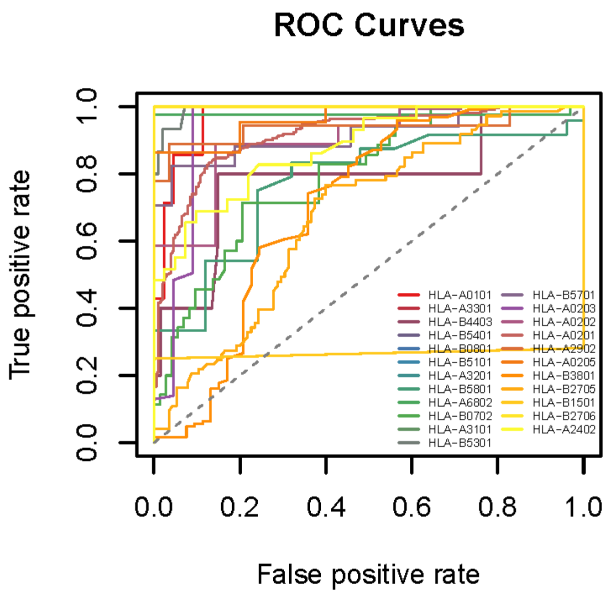

mhcflurry

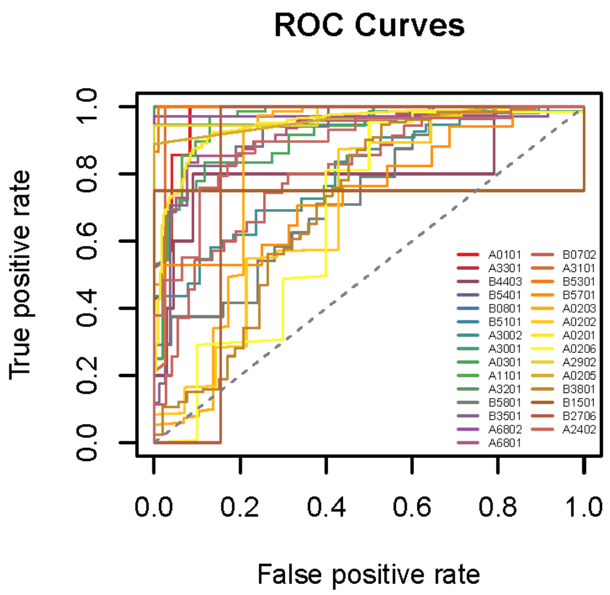

mhcflurry_pan

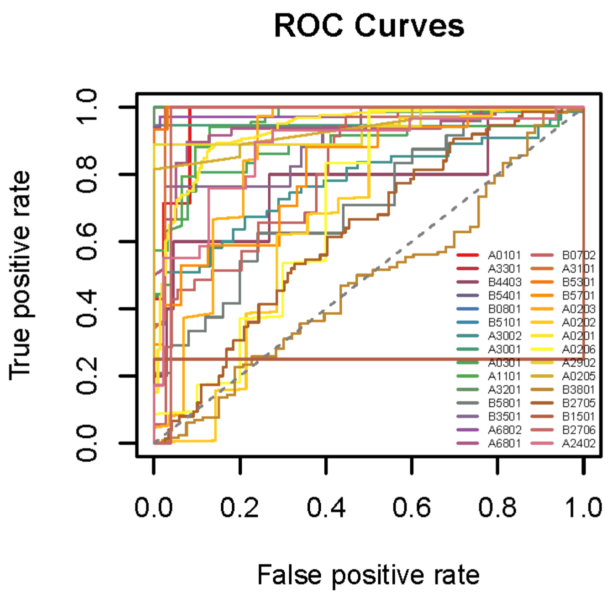

NetMHCpan4

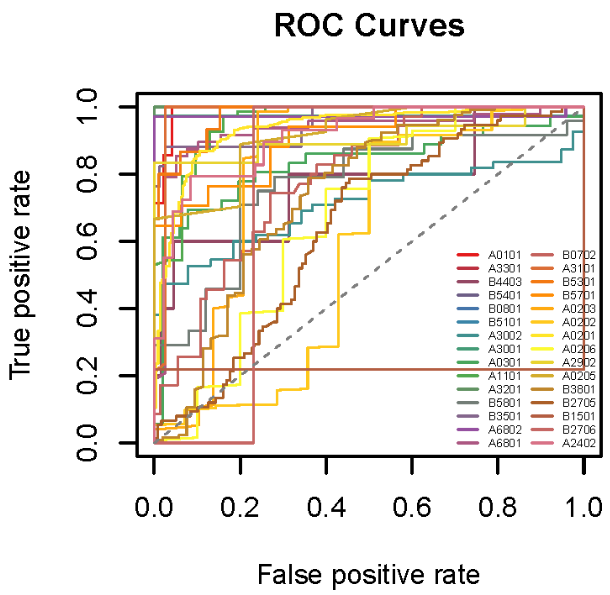

Public

MixMHCpred

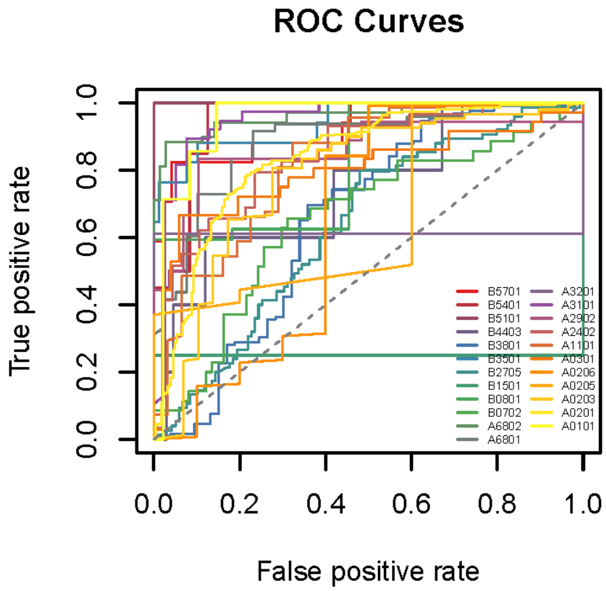

P Public
