# Supplementary figures and images for: Systematically benchmarking peptide-MHC binding predictors: From synthetic to naturally processed epitopes
Source: PLoS Comput Biol. 2018 Nov 8;14(11):e1006457. doi: 10.1371/journal.pcbi.1006457 (PMC6224037; doi:10.1371/journal.pcbi.1006457)

tepitope

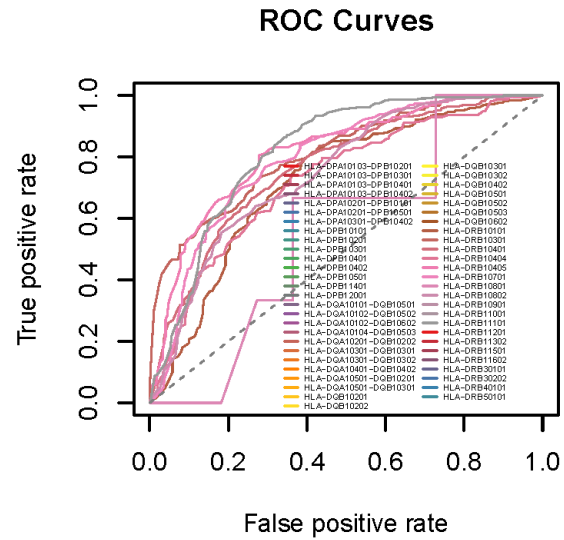

## ROC Curves

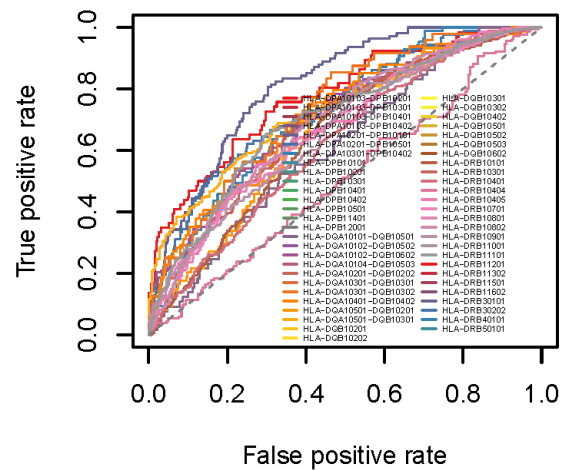

Supplement: S3 Fig — (PDF) [file pcbi.1006457.s005.pdf]
