# Supplementary material for: Systematically benchmarking peptide-MHC binding predictors: From synthetic to naturally processed epitopes
Source: PLoS Comput Biol. 2018 Nov 8;14(11):e1006457. doi: 10.1371/journal.pcbi.1006457 (PMC6224037; doi:10.1371/journal.pcbi.1006457)
Supplement: S4 Fig — (PDF) [file pcbi.1006457.s006.pdf]

Fig S4. **Additional data on the accuracy of MixMHCpred, NetMHC4 and NetMHCpan4 on predicting three MS-derived elution datasets.** (a) Boxplots showing the quartile distribution of normalized binding affinities (1-log10(IC50)/log10(50000)) predicted by NetMHC4, and HLA-ligand elution scores predicted by NetMHCpan4 and MixMHCpred, for MHC-eluted and non-eluted peptides in Dana Farber and Abelin datasets. Grey dashed line indicates predicted binder cutoff of 500 nM for NetMHC4 and predicted eluted peptide cutoff of 0.5 for NetMHCpan4 and MixMHCpred. (b) Boxplots showing quartile distribution of either normalized binding affinities (NetMHC4), elution scores (NetMHCpan4 and MixMHCpred), or percentile ranking scores (NetMHC4 and NetMHCpan4) for peptides in Bassani-Sternberg dataset. Different grey dashed lines were drawn accordingly depending predicted binding affinity, elution probability, or percentile rank. (c) FDr and FNr heatmap, calculated based on actual prediction score cutoff (binding affinity or elution probability) on three MS-derived datasets.

(a)

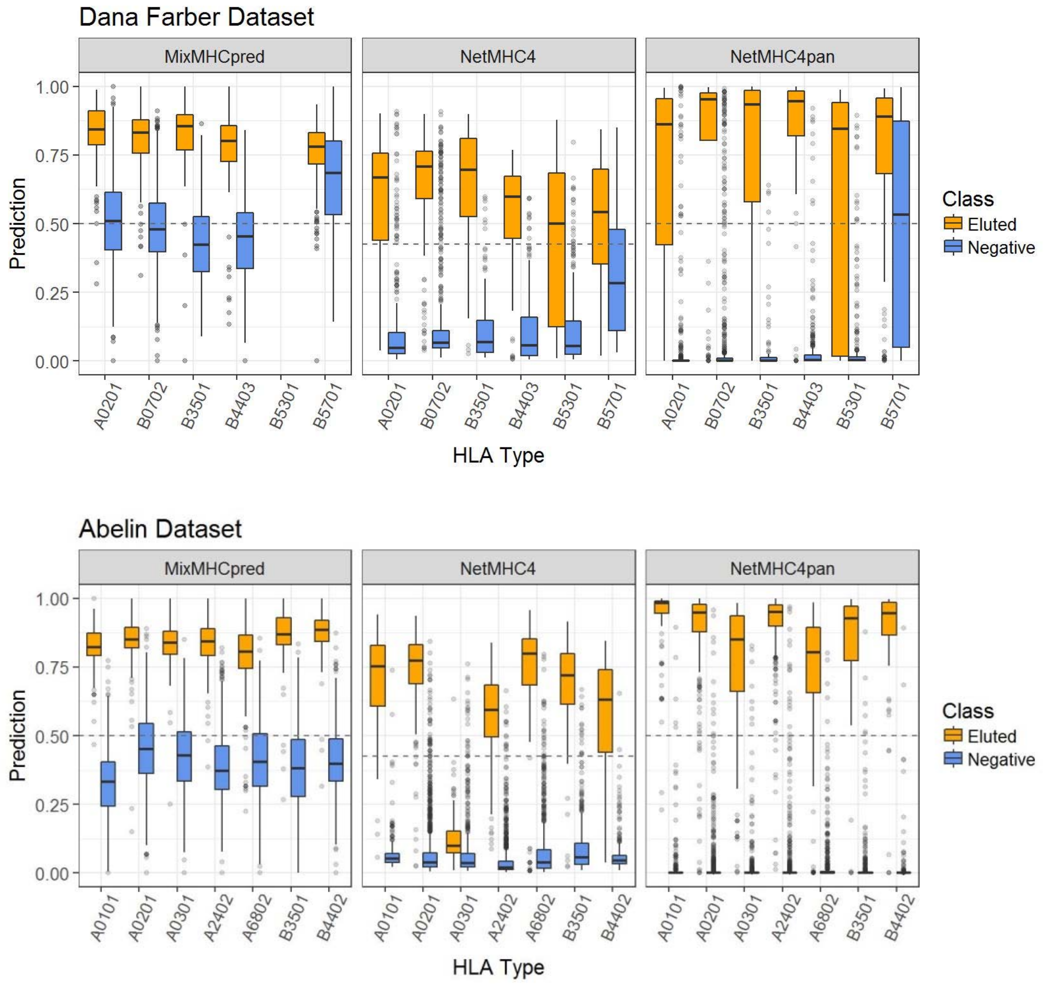

Public

(b)

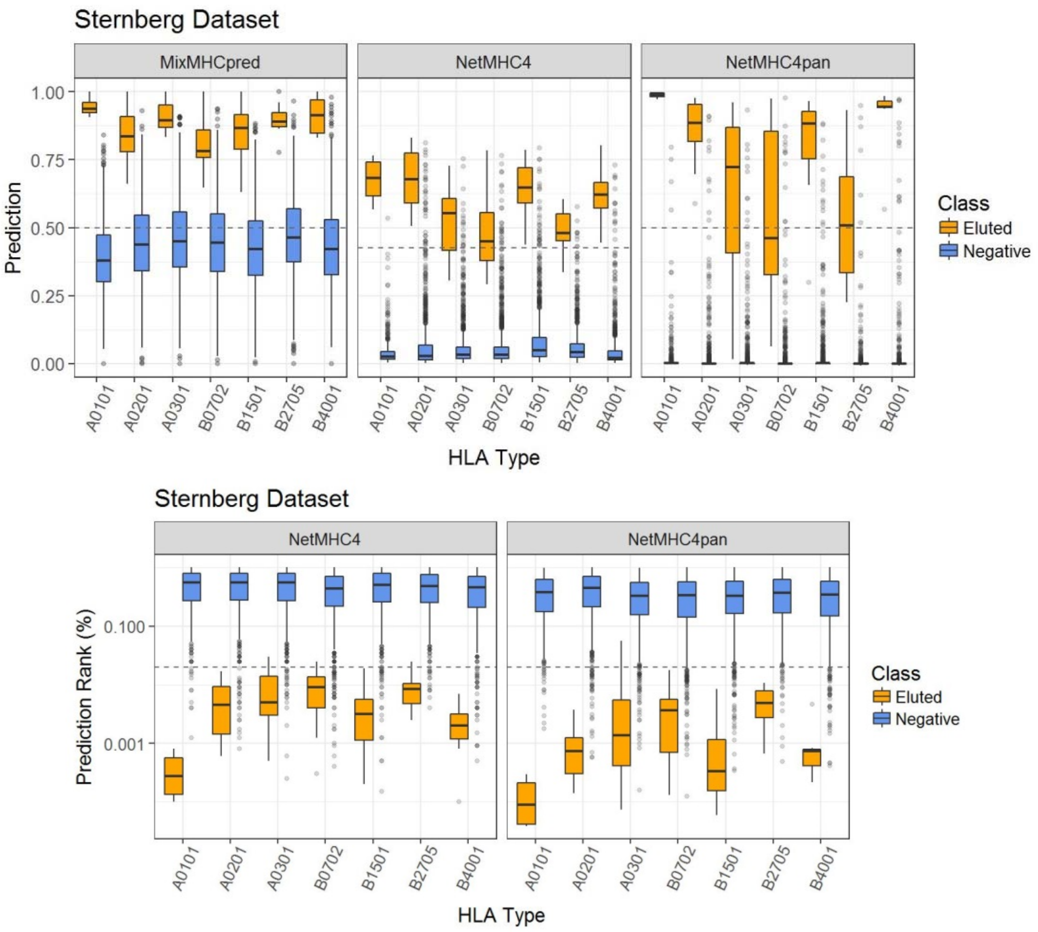

Sternberg Dataset
MixMHCpred
NetMHC4
NetMHC4pan
Prediction
1.00
0.75
0.50
0.25
0.00
A0101
A0201
A0301
B0702
B1501
B2705
B4001
HLA Type
Class
Eluted
Negative
Sternberg Dataset
NetMHC4
NetMHC4pan
Prediction Rank (%)
0.100
0.001
HLA Type
Class
Eluted
Negative

(c)

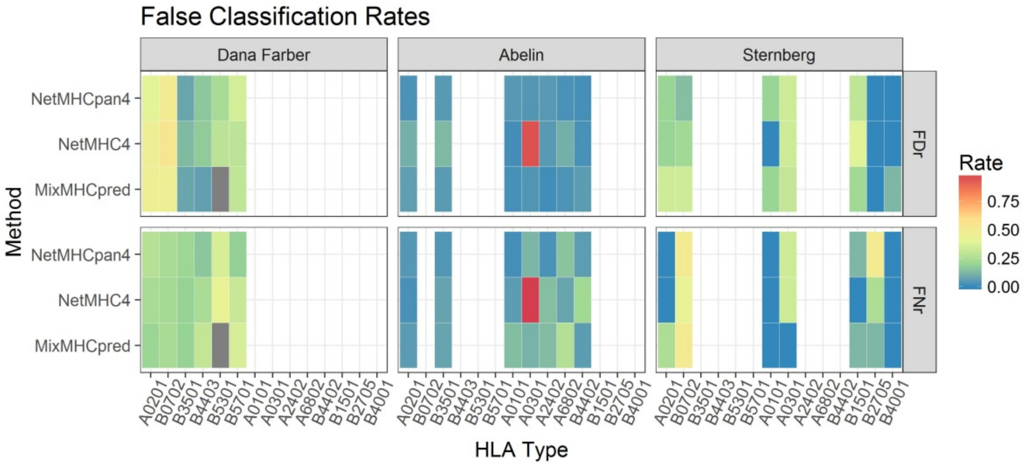

False Classification Rates
Dana Farber
Abelin
Sternberg
Method
NetMHCpan4
NetMHC4
MixMHCpred
FDr
FNr
Rate
0.75
0.50
0.25
0.00
A0201
B0702
B3501
B4403
B5301
B5701
A0101
A0301
A2402
A6802
B4402
B1501
B2705
B4001
HLA Type

Public
