# Supplementary material for: Systematically benchmarking peptide-MHC binding predictors: From synthetic to naturally processed epitopes
Source: PLoS Comput Biol. 2018 Nov 8;14(11):e1006457. doi: 10.1371/journal.pcbi.1006457 (PMC6224037; doi:10.1371/journal.pcbi.1006457)
Supplement: S5 Fig — (PDF) [file pcbi.1006457.s007.pdf]

Fig S5. **ROC curves and corresponding AUC values for predicting eluted *vs.* non-eluted MS-confirmed peptides in Dana Farber dataset**. All methods are for HLA class I prediction.

A0201

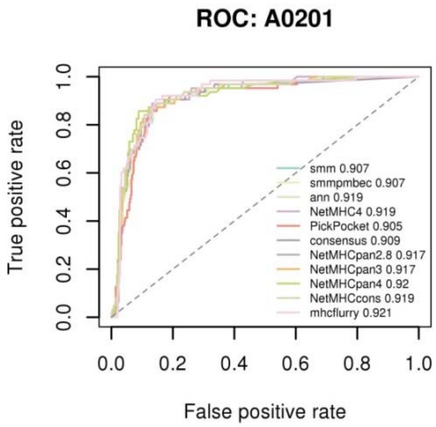


B0702

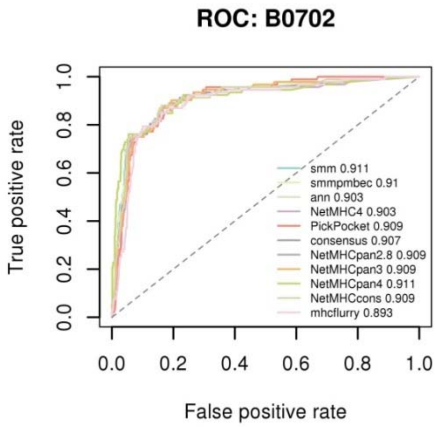


B3501

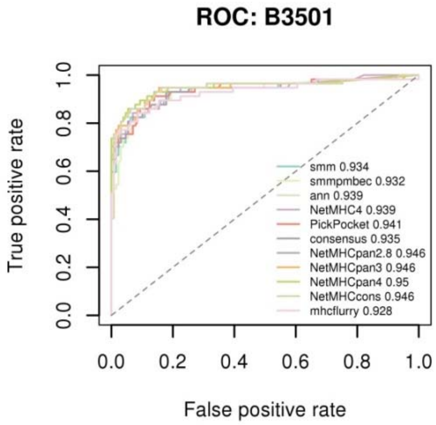


B4403

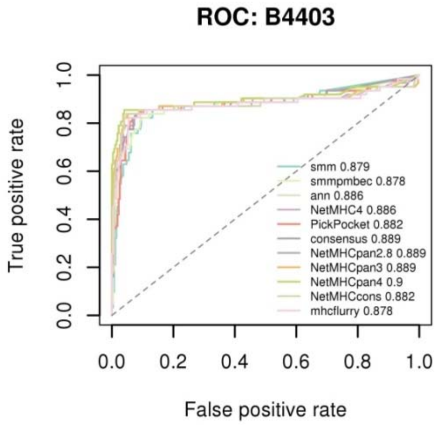


B5301

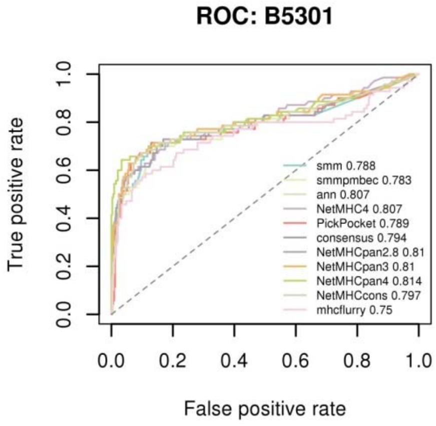


B5701

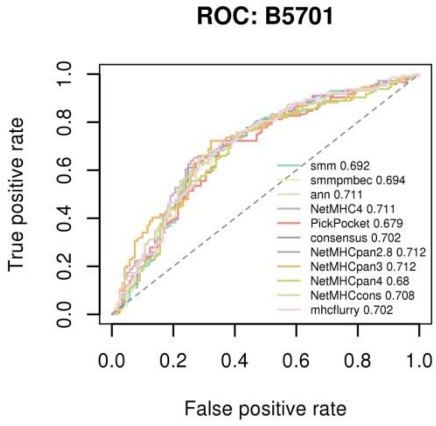


Public
