# Supplementary material for: Systematically benchmarking peptide-MHC binding predictors: From synthetic to naturally processed epitopes
Source: PLoS Comput Biol. 2018 Nov 8;14(11):e1006457. doi: 10.1371/journal.pcbi.1006457 (PMC6224037; doi:10.1371/journal.pcbi.1006457)
Supplement: S6 Fig — (PDF) [file pcbi.1006457.s008.pdf]

Fig S6. **ANN layer architecture of mhcflurry_pan.** ANN is deployed through keras and tensorflow packages. For activation function, *relu* and *sigmoid* were used for dense and output layers, correspondingly. For optimizer and loss function, adam and msd were used, respectively.

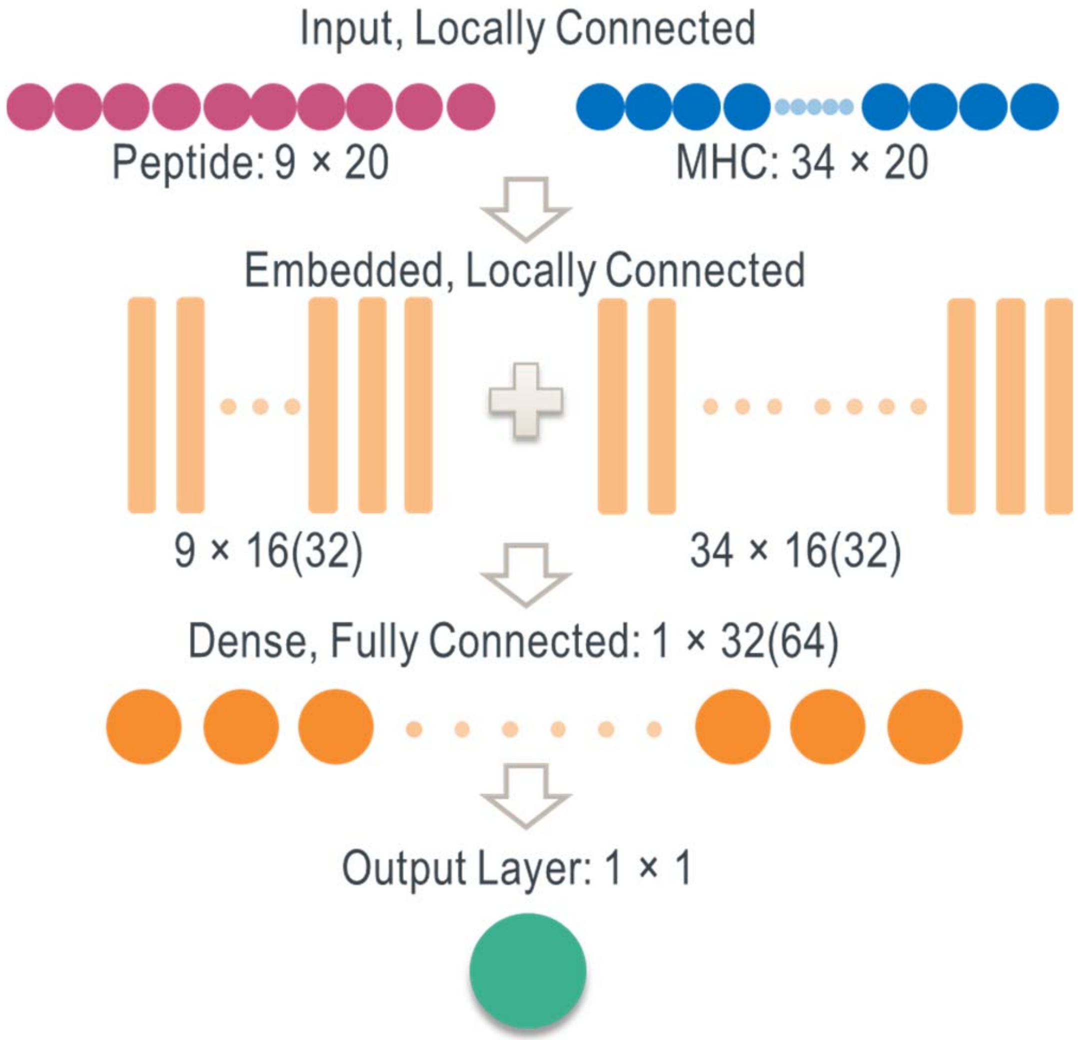

Public
